# Secretory Phospholipase A_2_-IIA and Cardiovascular Disease: A Mendelian Randomization Study

**DOI:** 10.1016/j.jacc.2013.06.044

**Published:** 2013-11-19

**Authors:** Michael V. Holmes, Tabassome Simon, Holly J. Exeter, Lasse Folkersen, Folkert W. Asselbergs, Montse Guardiola, Jackie A. Cooper, Jutta Palmen, Jaroslav A. Hubacek, Kathryn F. Carruthers, Benjamin D. Horne, Kimberly D. Brunisholz, Jessica L. Mega, Erik P.A. van Iperen, Mingyao Li, Maarten Leusink, Stella Trompet, Jeffrey J.W. Verschuren, G. Kees Hovingh, Abbas Dehghan, Christopher P. Nelson, Salma Kotti, Nicolas Danchin, Markus Scholz, Christiane L. Haase, Dietrich Rothenbacher, Daniel I. Swerdlow, Karoline B. Kuchenbaecker, Eleonora Staines-Urias, Anuj Goel, Ferdinand van 't Hooft, Karl Gertow, Ulf de Faire, Andrie G. Panayiotou, Elena Tremoli, Damiano Baldassarre, Fabrizio Veglia, Lesca M. Holdt, Frank Beutner, Ron T. Gansevoort, Gerjan J. Navis, Irene Mateo Leach, Lutz P. Breitling, Hermann Brenner, Joachim Thiery, Dhayana Dallmeier, Anders Franco-Cereceda, Jolanda M.A. Boer, Jeffrey W. Stephens, Marten H. Hofker, Alain Tedgui, Albert Hofman, André G. Uitterlinden, Vera Adamkova, Jan Pitha, N. Charlotte Onland-Moret, Maarten J. Cramer, Hendrik M. Nathoe, Wilko Spiering, Olaf H. Klungel, Meena Kumari, Peter H. Whincup, David A. Morrow, Peter S. Braund, Alistair S. Hall, Anders G. Olsson, Pieter A. Doevendans, Mieke D. Trip, Martin D. Tobin, Anders Hamsten, Hugh Watkins, Wolfgang Koenig, Andrew N. Nicolaides, Daniel Teupser, Ian N.M. Day, John F. Carlquist, Tom R. Gaunt, Ian Ford, Naveed Sattar, Sotirios Tsimikas, Gregory G. Schwartz, Debbie A. Lawlor, Richard W. Morris, Manjinder S. Sandhu, Rudolf Poledne, Anke H. Maitland-van der Zee, Kay-Tee Khaw, Brendan J. Keating, Pim van der Harst, Jackie F. Price, Shamir R. Mehta, Salim Yusuf, Jaqueline C.M. Witteman, Oscar H. Franco, J. Wouter Jukema, Peter de Knijff, Anne Tybjaerg-Hansen, Daniel J. Rader, Martin Farrall, Nilesh J. Samani, Mika Kivimaki, Keith A.A. Fox, Steve E. Humphries, Jeffrey L. Anderson, S. Matthijs Boekholdt, Tom M. Palmer, Per Eriksson, Guillaume Paré, Aroon D. Hingorani, Marc S. Sabatine, Ziad Mallat, Juan P. Casas, Philippa J. Talmud

**Affiliations:** 1 Faculty of Population Health Sciences, University College London, London, United Kingdom; 2 Assistance Publique–Hôpitaux de Paris (AP-HP), Hôpital Saint-Antoine, Department of Clinical Pharmacology, URC-EST, Paris, France; 3 Université Pierre et Marie Curie, Paris, France; INSERM, U 698, Paris, France; 4 Centre for Cardiovascular Genetics, Institute of Cardiovascular Science, University College London, London, United Kingdom; 5 Atherosclerosis Research Unit, Department of Medicine Solna, Karolinska Institutet, Stockholm, Sweden; 6 Center for Molecular Medicine, Karolinska University Hospital Solna, Stockholm, Sweden; 7 Department of Cardiology, Division Heart and Lungs, University Medical Center Utrecht, Utrecht, the Netherlands; 8 Julius Center for Health Sciences and Primary Care, University Medical Center Utrecht, the Netherlands; 9 Durrer Center for Cardiogenetic Research, Amsterdam, the Netherlands; 10 Unitat de Recerca en Lípids i Arteriosclerosi, IISPV, Universitat Rovira i Virgili, CIBERDEM, Reus, Spain; 11 Center for Experimental Medicine, Institute for Clinical and Experimental Medicine, Prague, Czech Republic; 12 Centre for Cardiovascular Science, University of Edinburgh, Edinburgh, Scotland, United Kingdom; 13 Intermountain Heart Institute, Intermountain Medical Center, Salt Lake City, Utah; 14 Department of Medicine, University of Utah School of Medicine, Salt Lake City, Utah; 15 TIMI Study Group, Divison of Cardiovascular Medicine, Brigham and Women's Hospital & Harvard Medical School, Boston, Massachusetts; 16 Durrer Center for Cardiogenetic Research, Amsterdam, the Netherlands; 17 Department of Clinical Epidemiology, Biostatistics and Bioinformatics, Academic Medical Center, University of Amsterdam, Amsterdam, the Netherlands; 18 Department of Biostatistics & Epidemiology, University of Pennsylvania School of Medicine, Philadelphia, Pennsylvania; 19 Division of Pharmacoepidemiology and Clinical Pharmacology, Utrecht Institute for Pharmaceutical Sciences, Utrecht University, Utrecht, the Netherlands; 20 Department of Cardiology, Leiden University Medical Center, Leiden, the Netherlands; 21 Department of Gerontology and Geriatrics, Leiden University Medical Center, Leiden, the Netherlands; 22 Department of Vascular Medicine, Academic Medical Center, University of Amsterdam, Amsterdam, the Netherlands; 23 Department of Epidemiology, Erasmus Medical Center, Rotterdam, the Netherlands; 24 Member of the Netherlands Consortium on Healthy Aging (NCHA), Leiden, the Netherlands; 25 Department of Cardiovascular Sciences, University of Leicester, Leicester, United Kingdom; 26 Leicester NIHR Biomedical Research Unit in Cardiovascular Disease, Glenfield Hospital, Leicester, United Kingdom; 27 Assistance Publique Hôpitaux de Paris, Hôpital Européen Georges Pompidou, Department of Cardiology, Paris, France; 28 Université Paris Descartes, Paris V, Paris, France; 29 Institute for Medical Informatics, Statistics and Epidemiology, University of Leipzig, Leipzig, Germany; 30 LIFE: Leipzig Research Center for Civilization Diseases, University of Leipzig, Leipzig, Germany; 31 Department of Clinical Biochemistry, Rigshospitalet, Copenhagen University Hospital, Copenhagen, Denmark; 32 Institute of Epidemiology and Medical Biometry, Ulm University, Ulm, Germany; 33 Division of Clinical Epidemiology & Aging Research, German Cancer Research Center, Heidelberg, Germany; 34 Centre for Cancer Genetic Epidemiology, Department of Public Health and Primary Care, University of Cambridge, Cambridge, United Kingdom; 35 Faculty of Epidemiology and Population Health, London School of Hygiene & Tropical Medicine, London, United Kingdom; 36 Wellcome Trust Centre for Human Genetics, University of Oxford, Oxford, United Kingdom; 37 Department of Cardiovascular Medicine, University of Oxford, Oxford, United Kingdom; 38 Division of Cardiovascular Epidemiology, Institute of Environmental Medicine, Karolinska Institutet, Stockholm, Sweden; 39 Cyprus Cardiovascular Educational and Research Trust, Nicosia, Cyprus and Cyprus International Institute for Environmental and Public Health in association with the Harvard School of Public Health, Cyprus University of Technology, Limassol, Cyprus; 40 Dipartimento di Scienze Farmacologiche e Biomolecolari, Universitá di Milano, Milan, Italy; 41 Centro Cardiologico Monzino, IRCCS, Milan, Italy; 42 Institute of Laboratory Medicine, Clinical Chemistry and Molecular Diagnostics, University Hospital Leipzig, Leipzig, Germany; 43 Department of Internal Medicine/Cardiology, Heart Center, University of Leipzig, Leipzig, Germany; 44 University Medical Center Groningen, University of Groningen, Department of Internal Medicine, Groningen, the Netherlands; 45 University Medical Center Groningen, University of Groningen, Department of Cardiology, Groningen, the Netherlands; 46 Department of Internal Medicine II-Cardiology, University of Ulm Medical Center, Ulm, Germany; 47 Cardiothoracic Surgery Unit, Department of Molecular Medicine and Surgery, Karolinska Institutet, Stockholm, Sweden; 48 Department for Nutrition and Health, National Institute for Public Health and the Environment, Bilthoven, the Netherlands; 49 Diabetes Research Group, Institute of Life Sciences, College of Medicine, Swansea University, Swansea, Wales, United Kingdom; 50 Department of Pathology and Medical Biology, Medical Biology Section, Molecular Genetics, University of Groningen, University Medical Center Groningen, Groningen, the Netherlands; 51 Inserm U970, Paris-Cardiovascular Research Center, Paris, France; 52 Department of Internal Medicine, Erasmus Medical Center, Rotterdam, the Netherlands; 53 Department of Vascular Medicine, University Medical Center Utrecht, Utrecht, the Netherlands; 54 Division of Population Health Sciences and Education, St George's, University of London, London, United Kingdom; 55 Leeds Institute of Genetics, Health and Therapeutics, University of Leeds, Leeds, United Kingdom; 56 Stockholm Heart Center, Stockholm, and Linköping University, Linkőping, Sweden; 57 Department of Cardiology, Academic Medical Center, University of Amsterdam, Amsterdam, the Netherlands; 58 Departments of Health Sciences & Genetics, University of Leicester, Leicester, United Kingdom; 59 Department of Vascular Surgery, Imperial College, London, United Kingdom; 60 Cyprus Cardiovascular Educational and Research Trust, Nicosia, Cyprus; 61 Institute of Laboratory Medicine, University Hospital Munich (LMU), Ludwig-Maximilians-University Munich, Munich, Germany; 62 MRC Centre for Causal Analyses in Translational Epidemiology (CAiTE), and Bristol Genetic Epidemiology Laboratories (BGEL), School of Social and Community Medicine, University of Bristol, Bristol, United Kingdom; 63 Robertson Centre for Biostatistics, University of Glasgow, Glasgow, Scotland, United Kingdom; 64 British Heart Foundation Glasgow Cardiovascular Research Centre, University of Glasgow, Glasgow, Scotland, United Kingdom; 65 Division of Cardiovascular Diseases, Department of Medicine, University of California San Diego, La Jolla, California; 66 VA Medical Center and University of Colorado School of Medicine, Denver, Colorado; 67 Department of Primary Care & Population Health, University College London, Royal Free Campus, London, United Kingdom; 68 Department of Public Health and Primary Care, University of Cambridge, Cambridge, United Kingdom; 69 Center for Applied Genomics, Children's Hospital of Philadelphia, Philadelphia, Pennsylvania; 70 Centre for Population Health Sciences, University of Edinburgh, United Kingdom; 71 Department of Clinical Epidemiology & Biostatistics, McMaster University, Hamilton, Ontario, Canada; 72 Department of Medicine, McMaster University, Hamilton, Ontario, Canada; 73 Interventional Cardiology, McMaster University, Hamilton, Ontario, Canada; 74 Population Health Research Institute, McMaster University and Hamilton Health Sciences, Hamilton, Ontario, Canada; 75 Interuniversity Cardiology Institute of the Netherlands, Utrecht, the Netherlands; 76 Department of Human Genetics, Leiden University Medical Center, Leiden, the Netherlands; 77 Preventive Cardiovascular Medicine, Penn Heart and Vascular Center, Philadelphia, Pennsylvania; 78 Division of Health Sciences, Warwick Medical School, University of Warwick, Coventry, United Kingdom; 79 Department of Pathology and Molecular Medicine, McMaster University, Hamilton, Ontario, Canada; 80 Genetic and Molecular Epidemiology Laboratory, McMaster University, Hamilton, Ontario, Canada; 81 Centre for Clinical Pharmacology, Division of Medicine, University College London, London, United Kingdom; 82 Division of Cardiovascular Medicine, University of Cambridge, Addenbrooke's Hospital, Cambridge, United Kingdom

**Keywords:** cardiovascular diseases, drug development, epidemiology, genetics, Mendelian randomization, ACS, acute coronary syndrome(s), CI, confidence interval, LDL-C, low-density lipoprotein cholesterol, MI, myocardial infarction, MVE, major vascular events, OR, odds ratio, RCT, randomized clinical trial, SNP, single-nucleotide polymorphism, sPLA_2_, secretory phospholipase A_2_

## Abstract

**Objectives:**

This study sought to investigate the role of secretory phospholipase A_2_ (sPLA_2_)-IIA in cardiovascular disease.

**Background:**

Higher circulating levels of sPLA_2_-IIA mass or sPLA_2_ enzyme activity have been associated with increased risk of cardiovascular events. However, it is not clear if this association is causal. A recent phase III clinical trial of an sPLA_2_ inhibitor (varespladib) was stopped prematurely for lack of efficacy.

**Methods:**

We conducted a Mendelian randomization meta-analysis of 19 general population studies (8,021 incident, 7,513 prevalent major vascular events [MVE] in 74,683 individuals) and 10 acute coronary syndrome (ACS) cohorts (2,520 recurrent MVE in 18,355 individuals) using rs11573156, a variant in *PLA2G2A* encoding the sPLA_2_-IIA isoenzyme, as an instrumental variable.

**Results:**

*PLA2G2A* rs11573156 C allele associated with lower circulating sPLA_2_-IIA mass (38% to 44%) and sPLA_2_ enzyme activity (3% to 23%) per C allele. The odds ratio (OR) for MVE per rs11573156 C allele was 1.02 (95% confidence interval [CI]: 0.98 to 1.06) in general populations and 0.96 (95% CI: 0.90 to 1.03) in ACS cohorts. In the general population studies, the OR derived from the genetic instrumental variable analysis for MVE for a 1-log unit lower sPLA_2_-IIA mass was 1.04 (95% CI: 0.96 to 1.13), and differed from the non-genetic observational estimate (OR: 0.69; 95% CI: 0.61 to 0.79). In the ACS cohorts, both the genetic instrumental variable and observational ORs showed a null association with MVE. Instrumental variable analysis failed to show associations between sPLA_2_ enzyme activity and MVE.

**Conclusions:**

Reducing sPLA_2_-IIA mass is unlikely to be a useful therapeutic goal for preventing cardiovascular events.

The secretory phospholipase A_2_ (sPLA_2_) enzymes, mostly comprising sPLA_2_-IIA, sPLA_2_-III, sPLA_2_-V, and sPLA_2_-X, hydrolyze phospholipids from the cell membrane surface and lipoproteins, producing pro-inflammatory lysophospholipids and eicosanoids (1). This activity may also modify low-density lipoprotein cholesterol (LDL-C) particles in the circulation increasing the binding of LDL-C onto blood vessel wall proteoglycans, promoting foam cell formation and the development of atherosclerosis (2). sPLA_2_-IIA is thought to be the most highly expressed of the sPLA_2_ enzymes (2) and its mass can be quantified specifically in plasma by enzyme-linked immunosorbent assay (3). In contrast, the assay for sPLA_2_ enzyme activity does not distinguish between the secretory isoenzymes IIA, III, V, and X (2).

Observational studies have indicated that higher circulating sPLA_2_-IIA mass and sPLA_2_ enzyme activity are associated with an increased risk of incident and recurrent MVE (comprising cardiovascular death, myocardial infarction [MI], and stroke) 3, 4, 5, 6, with the evidence being more compelling in primary prevention (4) than in patients with ACS (7). This suggests that sPLA_2_ isoenzymes, in particular IIA, may represent a novel therapeutic target for cardiovascular disease prevention. This hypothesis is supported by studies in mouse models that show over-expression of sPLA_2_-IIA associates with increased atherosclerotic lesion size (8).

Despite these encouraging findings, mechanistic studies in animals may not faithfully model the disease process in humans, and observational studies in humans cannot provide definitive evidence on causation. This is because higher sPLA_2_-IIA mass or sPLA_2_ enzyme activity may be a consequence not a cause of atherosclerosis or reflect an association with causal risk factors. An evaluation of sPLA_2_ as a therapeutic target is timely to help put into context the recent announcement that a phase III randomized trial (VISTA-16 [Vascular Inflammation Suppression to Treat Acute Coronary Syndrome for 16 Weeks] trial) (10) of varespladib, a first-in-class sPLA_2_ inhibitor (9) for major vascular event (MVE) prevention in patients with acute coronary syndrome (ACS), was stopped prematurely for lack of efficacy (11). Varespladib was designed to selectively block sPLA_2_-IIA, however, it also has minor inhibitory effects on other sPLA_2_ isoenzymes (Online Fig. 1) 12, 13.

## Methods

A total of 109,179 individuals of European descent from 36 studies were included in the analysis (Online Table 1), comprising 19 in general populations and 10 studies in patients with ACS. In addition, we included 4 case control studies of coronary artery disease, 1 cohort of patients with established arterial vascular disease or risk factors for cardiovascular disease (SMART [Second Manifestations of ARTerial disease] study), and 1 nested case-control study of coronary artery restenosis in patients with ACS undergoing percutaneous coronary intervention (GENDER [GENetic Determinants of Restenosis] study). These additional studies did not contribute toward the analyses set in general population or ACS studies, and were analyzed and reported separately (Online Appendix). Finally, tissue samples from 1 cohort of patients undergoing aortic valve surgery (ASAP [Advanced Study of Aortic Pathology]) were used to investigate the association of single-nucleotide polymorphisms (SNPs) in *PLA2G2A* with mRNA expression in liver, mammary artery, aorta, and heart with an external data source comprising 206 transplant donor liver samples used for replication (14). Approval from relevant ethical committees was obtained for collaborating studies. All analyses, unless otherwise stated, were performed using Stata 12.1 (StataCorp, College Station, Texas).

### Measurement of sPLA_2_-IIA mass and sPLA_2_ enzyme activity

sPLA_2_-IIA mass and sPLA_2_ enzyme activity were measured in 7 and 6 of the collaborating studies, respectively (Online Table 2). Assay methods are reported in Online Table 3. Owing to the time of blood sampling being greater than 1 month after the acute coronary event, samples for the KAROLA (Langzeiterfolge der KARdiOLogischen Anschlussheilbehandlung) study were not included in the analysis. The distributions of both sPLA_2_-IIA mass and sPLA_2_ enzyme activity were skewed, hence the traits were natural log(e) transformed prior to analysis.

### Observational analysis

We investigated correlations between log sPLA_2_-IIA mass and log sPLA_2_ enzyme activity in studies that measured both traits (Online Appendix, Online Fig. 2). To investigate the association between circulating sPLA_2_-IIA mass and sPLA_2_ enzyme activity with incident major vascular events in general populations, we used the European Prospective Investigation of Cancer (EPIC)-Norfolk study and to investigate the association with recurrent events in patients with ACS, we used 4 ACS cohorts (FAST-MI [French Registry of Acute ST-Elevation or Non–ST-elevation Myocardial Infarction], GRACE [Global Registry of Acute Coronary Events]-France, GRACE-Scotland, and MIRACL [Myocardial Ischemia Reduction with Acute Cholesterol Lowering] trial). For EPIC-Norfolk, the outcome was a composite of fatal and nonfatal MI, whereas for the 4 ACS cohorts, it was a composite of all-cause mortality or MI.

First, in the EPIC-Norfolk study we evaluated the cross-sectional correlates of sPLA_2_-IIA and sPLA_2_ enzyme activity with established and emerging risk factors using linear or logistic regression as appropriate. Second, we evaluated the shape of the association between sPLA_2_-IIA mass and sPLA_2_ enzyme activity with MI in the general population study, and with MI/all-cause mortality in the ACS cohorts. Third, we estimated the magnitude of the association per 1 log unit lower sPLA_2_-IIA mass and sPLA_2_ enzyme activity with MI or MI/all-cause mortality after statistical adjustment for potential confounders using logistic regression. Finally, we explored the independent effect of log sPLA_2_-IIA mass and log sPLA_2_ enzyme activity with MI or MI/all-cause mortality by fitting a logistic regression model that included both log sPLA_2_-IIA mass, and log sPLA_2_ enzyme activity in addition to potential confounders. The summary estimates were pooled across studies using fixed-effects meta-analysis. For full details of the observational analyses, please see the Online Appendix.

### Genetic analysis

All studies apart from the MIRACL trial contributed toward the genetic analysis (Online Table 2). Genotype coding was arranged to be directionally consistent with the effect of varespladib on sPLA_2_-IIA mass and sPLA_2_ enzyme activity. Traits that were nonnormally distributed (sPLA_2_-IIA mass, sPLA_2_ enzyme activity, C-reactive protein, triglycerides, and interleukin-6) were log(e) transformed, and differences between genotype groups were reported as a percentage difference.

### Selection of the genetic instrument and evaluation of its specificity and effect size

#### SNP Selection

Six tagging SNPs (15) that captured >90% of genetic variation in *PLA2G2A* in Europeans were evaluated in 3 studies (EPIC-Norfolk, GRACE-France, and UDACS [University College London Diabetes and Cardiovascular Study]). The rs11573156 variant that showed the lowest p value with sPLA_2_-IIA mass and sPLA_2_ enzyme activity (Online Fig. 3) was chosen for Mendelian randomization analysis. Rs11573156 was directly genotyped in all studies except 2 that imputed it and 1 that used a proxy SNP. Genotype frequencies were consistent across studies (Online Fig. 4) and did not deviate from Hardy-Weinberg equilibrium (at p < 0.001) (Online Table 4).

#### Specificity of Genetic Instrument for *PLA2G2A*

Affymetrix GeneChip Human Exon 1.0 ST expression arrays were used to quantify mRNA expression in the ASAP study, in which participants were genotyped using Illumina Human 610W-Quad Beadarray (including 101 SNPs in the region 200 kb upstream and downstream from the *PLA2G2A* locus). Please see the Online Appendix for further details of estimation of genotype association with mRNA expression.

#### Strength of Genetic Instrument (rs11573156 C>G) on sPLA_2_

We estimated the per C allele association between *PLA2G2A* rs11573156 and sPLA_2_ measures, as well as the proportion of variance (R^2^) of these measures explained by the rs11573156 variant.

#### Association Between Genetic Instrument and Putative and Established Cardiovascular Risk Factors

Twenty studies of individuals in which blood sampling occurred prior to the cardiovascular event were used to test the association of *PLA2G2A* rs11573156 (per C allele) with cardiovascular risk factors within each study using linear regression. Results were pooled using fixed (default) and random effects meta-analysis.

#### Cardiovascular Outcomes Examined

For the general population studies, MVE were separated into prevalent and incident, whereas for ACS cohorts, all events after recruitment were included and labeled as recurrent. Prevalent MVE were a composite of nonfatal MI and nonfatal stroke, and incident MVE were a composite of fatal/nonfatal MI and fatal/nonfatal stroke. For ACS cohorts, recurrent MVE were a composite of nonfatal MI, nonfatal stroke, and all-cause mortality. Individual components of MVE were also reported separately. See the Online Appendix for outcomes definitions per study and Online Table 5 for study contribution to the composite outcome.

#### Association Between Genetic Instrument and MVE

We conducted 2 genetic approaches: first, a genetic association analysis of the *PLA2G2A* rs11573156 variant with MVE, and; second, an instrumental variable analysis that quantified a causal effect per 1 log unit lower sPLA_2_-IIA mass and sPLA_2_ enzyme activity on MVE, under the assumptions of instrumental variable analysis (16). A total of 26 studies contributed to these 2 approaches, comprising 17 in general populations and 9 studies in patients with ACS.

For the genetic association approach, we estimated the within-study odds ratio (OR) per C allele of *PLA2G2A* rs11573156 with MVE using logistic regression and the results were pooled using fixed (default) and random-effects meta-analysis and used *I*^2^ to measure heterogeneity. All meta-analyses were stratified by clinical setting (general population or patients with ACS).

For the instrumental variable analysis, we first applied the pooled estimate of the gene variant on log sPLA_2_-IIA mass and log sPLA_2_ enzyme activity to each study, including studies that did not have measures of sPLA_2_-IIA mass or sPLA_2_ enzyme activity (17). An instrumental variable estimate was then generated (taking into account the uncertainty in both the gene-sPLA_2_ and gene-outcome associations) (18) for each study. The study-specific instrumental variable estimates were pooled using fixed-effects meta-analysis. Full details of the methodology are provided in the Online Appendix. We compared the instrumental variable estimates to the expected estimates based on the observational association between sPLA_2_-IIA mass, sPLA_2_ enzyme activity, and cardiovascular events.

### Treatment trials of varespladib

In order to contextualize the effect of the genetic instrument with the sPLA_2_ inhibitor (varespladib), we conducted a systematic review of RCTs (following PRISMA guidance) (19) to evaluate the effects of varespladib on sPLA_2_-IIA mass and other cardiovascular traits. To investigate the dose response between varespladib and sPLA_2_-IIA mass, we conducted a meta-regression analysis (for details, see the Online Appendix).

## Results

### Observational analysis of sPLA_2_-IIA mass and sPLA_2_ enzyme activity

**Figure 1:**
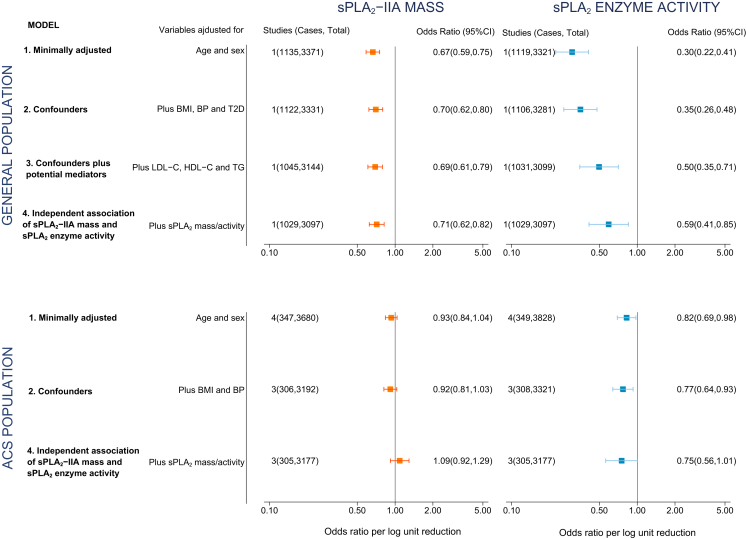
Association of a 1 Log Unit Lower sPLA_2_-IIA Mass or sPLA_2_ Enzyme Activity With Fatal/Nonfatal Myocardial Infarction in General Population Studies and All-Cause Mortality/Myocardial Infarction in Acute Coronary Syndrome Studies The general population study was EPIC-Norfolk and the 4 acute coronary syndrome cohorts were FAST-MI (French Registry of Acute ST-Elevation or Non–ST-elevation Myocardial Infarction), GRACE (Global Registry of Acute Coronary Events)-France, GRACE-Scotland, and MIRACL (Myocardial Ischemia Reduction with Acute Cholesterol Lowering). In Model 1, only age and gender were introduced as covariates. We then additionally adjusted for covariates (blood pressure [BP], body mass index [BMI], type 2 diabetes [T2D]) that could confound the association between secretory phospholipase A_2_ (sPLA_2_) and coronary heart disease (CHD; Model 2). Because lipids may mediate the association between sPLA_2_-IIA and CHD, we did not include lipids in Model 2, but included them in Model 3 (only available in the general population cohort). Finally, to investigate whether there was an independent association between sPLA_2_-IIA mass **(orange)**, sPLA_2_ enzyme activity **(blue)**, and CHD, we additionally included sPLA_2_ enzyme activity where sPLA_2_-IIA mass was the explanatory variable (and vice-versa; Model 4). CI = confidence interval; HDL-C = high-density lipoprotein cholesterol; LDL-C = low-density lipoprotein cholesterol; OR = odds ratio; TG = triglycerides.

Lower levels of sPLA_2_-IIA mass and lower levels of sPLA_2_ enzyme activity each were associated with a reduced risk of cardiovascular events in the general population with an OR for fatal/nonfatal MI of 0.67 (95% confidence interval [CI]: 0.59 to 0.75) and 0.30 (95% CI: 0.22 to 0.41) per 1 log unit lower sPLA_2_-IIA mass and sPLA_2_ enzyme activity, respectively, after adjustment for age and sex (Fig. 1). For studies set in ACS, the corresponding summary ORs for all-cause mortality or MI were 0.93 (95% CI: 0.84 to 1.04) and 0.82 (95% CI: 0.69 to 0.98), respectively (Fig. 1). The log-linear model provided the best fit (p ≥ 0.1 for a quadratic model in all comparisons) (Online Table 6), indicating a constant proportional decrease in the relative odds per 1 log unit lower sPLA_2_-IIA mass or sPLA_2_ enzyme activity.

sPLA_2_-IIA mass and sPLA_2_ enzyme activity associated with several established and emerging cardiovascular risk factors in the general population (Online Tables 7 and 8). In general, adjustment for cardiovascular risk factors diminished the association between sPLA_2_-IIA mass and sPLA_2_ enzyme activity with incident MI in the general population, though the association persisted following multivariate adjustment (Fig. 1). Interestingly, both associations (sPLA_2_-IIA mass and sPLA_2_ enzyme activity with MI) remained after adjustment for one another. For ACS cohorts, only sPLA_2_ enzyme activity was associated with recurrent events (Fig. 1).

### Selection and validation of the genetic instrument for sPLA_2_-IIA

rs11573156 C>G showed the lowest p value with sPLA_2_-IIA mass (p = 5.49 × 10^–180^) and sPLA_2_ enzyme activity (p = 3.29 × 10^–5^) and was prioritized for analysis in the remaining studies (Online Fig. 3).

**Figure 2:**
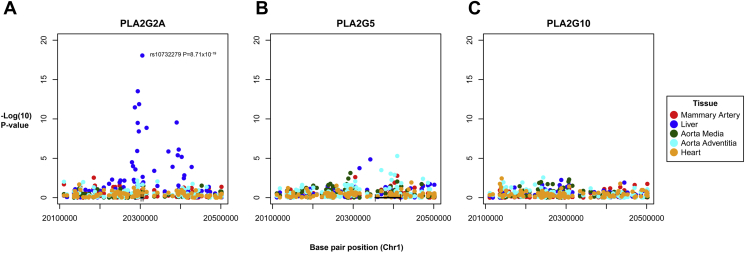
Association of SNPs in the *PLA2G2A* Region With mRNA Expression Manhattan plots of single-nucleotide polymorphisms (SNPs) in the *PLA2G2A* region and association with mRNA expression of **(A)***PLA2G2A*, **(B)***PLA2G5*, and **(C)***PLA2G10*, color-coded by tissue type. The p values for the association between rs10732279 and mRNA expression of *PLA2G5* or *PLA2G10* were 0.04 and 0.88, respectively.

To evaluate the specificity of the rs11573156 variant for sPLA_2_-IIA, we analyzed the association of SNPs in *PLA2G2A* with mRNA expression of 3 different sPLA_2_ isoenzymes, encoded by distinct genes (*PLA2G2A* for sPLA_2_-IIA and *PLA2G5* for sPLA_2_-V in close proximity on chromosome 1, and *PLA2G10* for sPLA_2_-X on chromosome 10). *PLA2G2A* was mainly expressed in the liver, aortic adventitia and heart (Online Fig. 5). The SNP showing strongest association with *PLA2G2A* mRNA expression in liver was rs10732279A>G (p = 8.71 × 10^–19^) (Fig. 2A), in strong linkage disequilibrium with rs11573156 (R^2^ = 0.91 in Europeans, HapMap release 21) and explained 31% of the variance of *PLA2G2A* mRNA. These findings were replicated in an external data source comprising 206 transplant donor liver samples (p = 4.76 × 10^–8^) (14). rs10732279 showed no association with either *PLA2G5* or *PLA2G10* mRNA expression confirming the specificity of the genetic instrument for sPLA_2_-IIA (Figs. 2B and 2C).

### Association of rs11573156 with sPLA_2_-IIA mass and sPLA_2_ enzyme activity

**Figure 3:**
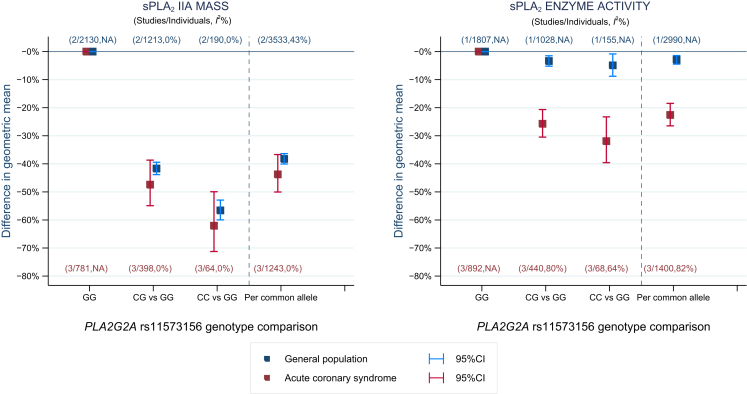
Meta-Analysis Pooled Estimates of the Association Between *PLA2G2A* rs11573156 With sPLA_2_-IIA Mass and sPLA_2_ Enzyme Activity The analysis is separated by study setting into general populations (EPIC [European Prospective Investigation of Cancer]-Norfolk, UDACS [University College London Diabetes and Cardiovascular Study]; **blue**) and acute coronary syndrome (FAST-MI [French Registry of Acute ST-Elevation or Non–ST-elevation Myocardial Infarction], GRACE [Global Registry of Acute Coronary Events]-France, GRACE-Scotland; **red**). The percentage estimate was obtained by back-transforming the *PLA2G2A* rs11573156 log sPLA_2_ association to obtain the relative difference, which was converted to a percentage by subtracting 1 from the relative difference and multiplying the fraction by 100. NA = not applicable either because there were too few studies (<3 studies) to synthesize an *I*^2^ estimate, or the value could not be calculated for the reference genotype group (GG). sPLA_2_ = secretory phospholipase A_2_.The 3 genotype groups for the rs11573156 SNP are: 1) GG = reference group; 2) CG: 1 copy of the sPLA_2_-lowering (common) C-allele; 3) CC: 2 copies of the sPLA_2_-lowering C-allele.

In 3 studies of 1,400 individuals with ACS and 2 general population studies of 3,533 individuals, an allele dose-dependent association was observed between rs11573156 and sPLA_2_-IIA mass and sPLA_2_ enzyme activity (Fig. 3). For each additional C allele of rs11573156, sPLA_2_-IIA mass was lower by 38% (95% CI: 36% to 40%) in studies of general populations and 44% (95% CI: 37% to 50%) in studies of ACS patients, compared with individuals homozygous for the G allele. The proportion of variance of sPLA_2_-IIA mass explained by rs11573156 in general population and ACS studies was 21% (95% CI: 18% to 23%) and 6% (95% CI: 3% to 9%), respectively.

In contrast, the effect of the rs11573156 C allele on sPLA_2_ enzyme activity was smaller and varied considerably by study setting, with a 3% reduction (95% CI: 1% to 5%) in studies of general populations and 23% reduction (95% CI: 19% to 27%) for studies of ACS patients. The proportion of variance of sPLA_2_ enzyme activity explained by rs11573156 was 0.5% (95% CI: 0.0% to 1.0%) and 3% (95% CI: 1% to 5%) in the general population and ACS cohorts, respectively.

We identified no major associations between rs11573156 and blood pressure, lipid fractions, inflammation markers, or carotid intima-media thickness (Online Tables 9 to 11).

### Comparison of pharmacological modification of sPLA_2_ in randomized clinical trials and carriage of the PLA2G2A variant in populations

We identified 4 randomized clinical trials (RCTs) of the sPLA_2_ inhibitor varespladib in a total of 1,300 individuals (Online Fig. 6, Online Table 12) 9, 20, 21, 22. A meta-regression suggested varespladib treatment produced a dose-dependent reduction in sPLA_2_-IIA mass (p for meta-regression = 0.06) (Online Fig. 7). The most frequently studied dose of varespladib (500 mg/day) reduced sPLA_2_-IIA mass by 78% (95% CI: 62% to 94%). The effect of varespladib on sPLA_2_ enzyme activity was not reported in RCTs because activity was reported to be beneath the lower limit of quantification of the assay 20, 21, 22.

### Association between rs11573156 and MVE

**Figure 4:**
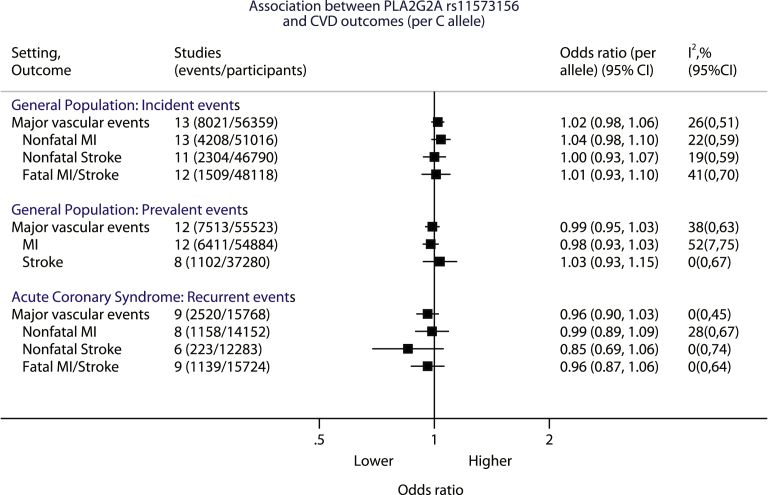
Meta-Analysis Pooled Estimates of the Association Between *PLA2G2A* rs11573156 and MVE (Including Individual Components) Stratified by Clinical Setting Into General Population and ACS Patients Each plot represents the *PLA2G2A* rs11573156 per C allele odds ratio, with genotype grouping arranged to mimic the effects of pharmacological lowering of secretory phospholipase A_2_ (sPLA_2_)-IIA (i.e., if lowering of sPLA_2_-IIA mass were to reduce risk of cardiovascular events, the odds ratio in the plot should be <1). Major vascular events (MVE) comprise fatal/nonfatal MI or stroke in general population studies and fatal/nonfatal MI or stroke or all-cause mortality in ACS studies. CI = confidence interval. Fatal myocardial infarction (MI)/stroke included all-cause mortality for some acute coronary syndrome (ACS) studies (see Online Table 5 for further details).

In a meta-analysis across 13 population studies (8,021 incident events in 56,359 individuals), there was no association between the C allele of rs11573156 and incident MVE (OR: 1.02; 95% CI: 0.98 to 1.06), nor with any of the individual components (Fig. 4, Online Fig. 8). Similarly, in 12 studies with 7,513 prevalent events in 55,523 individuals, there was no association between the rs11573156 C allele with prevalent MVE (OR: 0.99; 95% CI: 0.95 to 1.03), or with any of the individual components (Fig. 4, Online Fig. 9). For the 9 ACS studies with 2,520 recurrent events in 15,768 patients, there was also no association between the C allele of rs11573156 and recurrent MVE (OR: 0.96; 95% CI: 0.90 to 1.03) (Fig. 4, Online Fig. 10). Similar findings were obtained using a random-effects model for meta-analysis (Online Table 13).

### Extreme genotype comparison

Individuals homozygous for the rs11573156 C allele had a 57% to 62% lower sPLA_2_-IIA mass compared with those homozygous for the G allele (Fig. 3), which was similar in magnitude to the 78% reduction seen with 500 mg/day varespladib dose used in VISTA-16. Using this genotype comparison, a null effect was again observed for MVE in all clinical settings: incident (5,175 cases; OR: 0.99; 95% CI: 0.89 to 1.10), prevalent (3,545 cases; OR: 1.00; 95% CI: 0.88 to 1.13), and recurrent (1,626 cases; OR 0.89; 95% CI: 0.74 to 1.06).

### Instrumental variable analysis of sPLA_2_ on MVE

For the general population studies, instrumental variable analysis showed a null effect between sPLA_2_-IIA mass and incident MVE (OR per 1 log unit lower sPLA_2_-IIA mass: 1.04; 95% CI: 0.96 to 1.13) that was in contrast to the expected association based on observational analysis (OR: 0.69; 95% CI: 0.61 to 0.79). Similarly, for sPLA_2_ enzyme activity, observational studies showed an OR of 0.50 (95% CI: 0.35 to 0.71), yet null associations were obtained for the instrumental variable estimates for sPLA_2_ enzyme activity and incident MVE (OR: 1.87; 95% CI: 0.47 to 7.49), although the CIs were wide due to the weak effect of the rs11573156 variant on sPLA_2_ enzyme activity in the general population.

**Figure 5:**
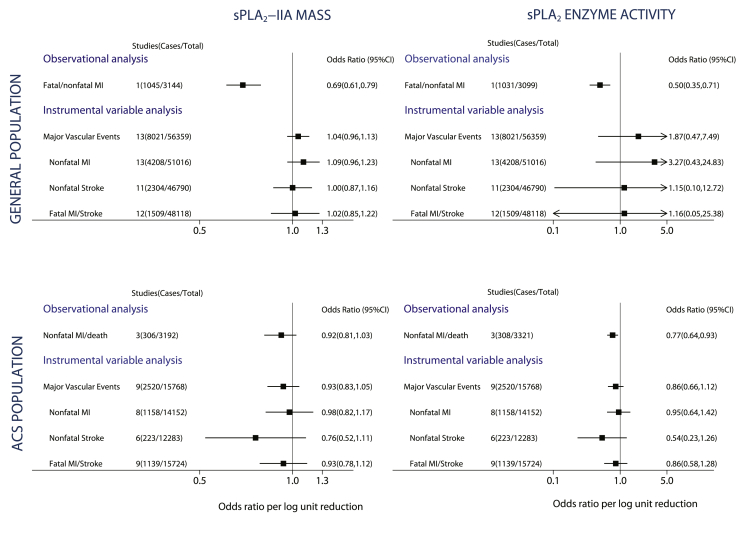
Observational and Instrumental Variable Estimates Per 1 Log Unit Lower sPLA_2_-IIA Mass and sPLA_2_ Enzyme Activity With Major Vascular Events The observational analyses were adjusted for age, sex, BMI, BP, T2D, LDL-C, HDL-C, and TG in general population cohorts (corresponding to Model 3 in Fig. 1) and for age, sex, BMI, and BP in ACS cohorts (corresponding to Model 2 in Fig. 1). Abbreviations as in Figure 1.

For the ACS studies, the instrumental variable estimate for sPLA_2_-IIA mass and recurrent MVE was also null (OR per 1 log unit lower sPLA_2_-IIA mass: 0.93; 95% CI: 0.83 to 1.05) and consistent with the observational estimate (OR: 0.92; 95% CI: 0.81 to 1.03). For sPLA_2_ enzyme activity, no association was identified for the instrumental variable estimate with MVE (OR: 0.86, 95% CI: 0.66 to 1.12), which was similar to the observational estimate (OR: 0.77, 95% CI: 0.64 to 0.93) (Fig. 5).

## Discussion

We used a genetic approach to judge the causal role of sPLA_2_-IIA on MVE and, by extension, to evaluate if inhibition of sPLA_2_-IIA represents a valid therapeutic target for cardiovascular prevention. We identified a SNP in *PLA2G2A* (rs11573156) that had a large and specific effect on circulating sPLA_2_-IIA mass and a small-to-modest effect on sPLA_2_ enzyme activity, but found no association between rs11573156 and incident, prevalent or recurrent MVE. This study provides evidence that the observational association between sPLA_2_-IIA mass and MVE is likely due to residual confounding or reverse causality.

Our Mendelian randomization analysis used a single genetic instrument that had a remarkable effect on sPLA_2_-IIA mass, explaining between 6% and 21% of its variance, a value several times higher than that observed for all genome wide association studies hits on blood pressure (1% for 29 SNPs combined) (23) and similar to that for LDL-C (∼12% for 49 SNPs combined) (24). The strength of our genetic instrument together with the large sample size analyzed strongly support the instrumental variable estimates that indicate a null effect of sPLA_2_-IIA mass with cardiovascular events.

The key SNP in our study (rs11573156) had a smaller impact on sPLA_2_ enzyme activity than sPLA_2_-IIA mass, in particular for general population studies. Because sPLA_2_ enzyme activity is a composite of several sPLA_2_ isoenzymes (2), it is not surprising that a *PLA2G2A* SNP (specific for sPLA_2_-IIA) explained a smaller proportion of variance of sPLA_2_ enzyme activity compared with sPLA_2_-IIA mass.

While this manuscript was being prepared, a phase III RCT of varespladib (VISTA-16) (10) was halted for lack of efficacy (11). VISTA-16 was to enroll up to 6,500 patients with ACS and randomize them to 500 mg/day varespladib or placebo for 16 weeks with a primary combined endpoint of cardiovascular death, nonfatal MI, nonfatal stroke, or documented unstable angina. We hypothesize that the null findings from our Mendelian randomization analysis may provide an eventual explanation for the lack of efficacy of varespladib in VISTA-16.

We did not find an association between lower sPLA_2_-IIA mass and lower rates of recurrent MVE in ACS patients, unlike earlier reports (10). With CIs that span ORs from 0.81 to 1.03, we cannot rule out a false negative finding due to a limited number of events. Alternatively, initial studies often overestimate the effect of a biomarker on a health outcome, and as more evidence accrues, the magnitude of the effect may diminish and in some cases disappears, known as the “winner's curse.”

### Study limitations

First, we did not have data from a common set of participants with all 3 key variables: sPLA_2_ measures, genetic information, and cardiovascular events. This is a common scenario with large-scale meta-analyses of Mendelian randomization studies that include novel biomarkers (25), but the instrumental variable approach helps overcome this problem. Second, given the impact of the SNP on sPLA_2_ enzyme activity was more modest than its effect on sPLA_2_-IIA mass, our genetic analyses do not exclude a possible causal role of other isoforms such as sPLA_2_-III, -V, and -X in cardiovascular disease. However, our genetic data do provide clear evidence against a causal role of sPLA_2_-IIA mass in incident MVE in the general population. With regard to an ACS population, our analysis includes 2,520 recurrent MVE in patients with ACS, which is 6-fold greater than the 385 primary events that VISTA-16 intended to accrue (10). Furthermore, comparing individuals homozygous for the rs11573156 C allele to those homozygous for the G allele resulted in a reduction in sPLA_2_-IIA mass similar to the effect of varespladib 500 mg/day and also showed no association with MVE.

## Conclusions

Our large-scale Mendelian randomization analysis suggests that sPLA_2_-IIA is unlikely to be a valid therapeutic target for prevention of cardiovascular events. The concordance of our genetic findings with the lack of efficacy of the phase III varespladib trial supports the wider use of this type of genetic approach earlier in drug development to prioritize which drug targets to take through to RCTs.

## References

[1] Rosenson R.S., Hurt-Camejo E. (2012). Phospholipase A2 enzymes and the risk of atherosclerosis. Eur Heart J.

[2] Mallat Z., Lambeau G., Tedgui A. (2010). Lipoprotein-associated and secreted phospholipases A in cardiovascular disease: roles as biological effectors and biomarkers. Circulation.

[3] Mallat Z., Steg P.G., Benessiano J. (2005). Circulating secretory phospholipase A2 activity predicts recurrent events in patients with severe acute coronary syndromes. J Am Coll Cardiol.

[4] Boekholdt S.M., Keller T.T., Wareham N.J. (2005). Serum levels of type II secretory phospholipase A2 and the risk of future coronary artery disease in apparently healthy men and women: the EPIC-Norfolk Prospective Population Study. Arterioscler Thromb Vasc Biol.

[5] Mallat Z., Benessiano J., Simon T. (2007). Circulating secretory phospholipase A2 activity and risk of incident coronary events in healthy men and women: the EPIC-Norfolk study. Arterioscler Thromb Vasc Biol.

[6] O'Donoghue M.L., Mallat Z., Morrow D.A. (2011). Prognostic utility of secretory phospholipase A(2) in patients with stable coronary artery disease. Clin Chem.

[7] Ryu S.K., Mallat Z., Benessiano J. (2012). Phospholipase A2 enzymes, high-dose atorvastatin, and prediction of ischemic events after acute coronary syndromes. Circulation.

[8] Tietge U.J., Pratico D., Ding T. (2005). Macrophage-specific expression of group IIA sPLA2 results in accelerated atherogenesis by increasing oxidative stress. J Lipid Res.

[9] Dzavik V., Lavi S., Thorpe K. (2010). The sPLA2 Inhibition to Decrease Enzyme Release after Percutaneous Coronary Intervention (SPIDER-PCI) trial. Circulation.

[10] Nicholls S.J., Cavender M.A., Kastelein J.J. (2012). Inhibition of secretory phospholipase A(2) in patients with acute coronary syndromes: rationale and design of the Vascular Inflammation Suppression to Treat Acute Coronary Syndrome for 16 Weeks (VISTA-16) Trial. Cardiovasc Drugs Ther.

[11] Clinical Study Report for Study AN-CVD2233. Evaluation of the Safety and Efficacy of Short-Term A-002 Treatment in Subjects with Acute Coronary Syndromes. 2012. Available at: http://www.anthera.com/VISTA-16.pdf. Accessed September 2013.

[12] Snyder D.W., Bach N.J., Dillard R.D. (1999). Pharmacology of LY315920/S-5920, [[3-(aminooxoacetyl)-2-ethyl-1- (phenylmethyl)-1H-indol-4-yl]oxy] acetate, a potent and selective secretory phospholipase A2 inhibitor: a new class of anti-inflammatory drugs, SPI. J Pharmacol Exp Ther.

[13] Fraser H., Hislop C., Christie R.M. (2009). Varespladib (A-002), a secretory phospholipase A2 inhibitor, reduces atherosclerosis and aneurysm formation in ApoE-/- mice. J Cardiovasc Pharmacol.

[14] Innocenti F., Cooper G.M., Stanaway I.B. (2011). Identification, replication, and functional fine-mapping of expression quantitative trait loci in primary human liver tissue. PLoS Genet.

[15] Wootton P.T., Drenos F., Cooper J.A. (2006). Tagging-SNP haplotype analysis of the secretory PLA2IIa gene PLA2G2A shows strong association with serum levels of sPLA2IIa: results from the UDACS study. Hum Mol Genet.

[16] Lawlor D.A., Harbord R.M., Sterne J.A., Timpson N. (2008). Davey Smith G. Mendelian randomization: using genes as instruments for making causal inferences in epidemiology. Stat Med.

[17] Thompson J.R., Minelli C., Abrams K.R., Tobin M.D., Riley R.D. (2005). Meta-analysis of genetic studies using Mendelian randomization—a multivariate approach. Stat Med.

[18] Thomas D.C., Lawlor D.A., Thompson J.R. (2007). Re: estimation of bias in nongenetic observational studies using “Mendelian triangulation” by Bautista et al. Ann Epidemiol.

[19] Moher D., Liberati A., Tetzlaff J., Altman D.G. (2009). Preferred reporting items for systematic reviews and meta-analyses: the PRISMA statement. PLoS Med.

[20] Rosenson R.S., Hislop C., Elliott M., Stasiv Y., Goulder M., Waters D. (2010). Effects of varespladib methyl on biomarkers and major cardiovascular events in acute coronary syndrome patients. J Am Coll Cardiol.

[21] Rosenson R.S., Hislop C., McConnell D. (2009). Effects of 1-H-indole-3-glyoxamide (A-002) on concentration of secretory phospholipase A2 (PLASMA study): a phase II double-blind, randomised, placebo-controlled trial. Lancet.

[22] Rosenson R.S., Elliott M., Stasiv Y., Hislop C. (2011). Randomized trial of an inhibitor of secretory phospholipase A2 on atherogenic lipoprotein subclasses in statin-treated patients with coronary heart disease. Eur Heart J.

[23] Padmanabhan S., Newton-Cheh C., Dominiczak A.F. (2012). Genetic basis of blood pressure and hypertension. Trends Genet.

[24] Teslovich T.M., Musunuru K., Smith A.V. (2010). Biological, clinical and population relevance of 95 loci for blood lipids. Nature.

[25] Minelli C., Thompson J.R., Tobin M.D., Abrams K.R. (2004). An integrated approach to the meta-analysis of genetic association studies using Mendelian randomization. Am J Epidemiol.

