# Supplementary material for: Secretory Phospholipase A2-IIA and Cardiovascular Disease: A Mendelian Randomization Study
Source: J Am Coll Cardiol. 2013 Nov 19;62(21):1966–76. doi: 10.1016/j.jacc.2013.06.044 (PMC3826105; doi:10.1016/j.jacc.2013.06.044)
Supplement: Online Data [file mmc1.doc]

## Table 1. Studies contributing towards the collaboration

| **Study** | **Study design** | **Geographical location** | **Sampling frame** | **Participants included** | **Baseline year(s)** | **% Female** | **Age, mean (SD)** |
| --- | --- | --- | --- | --- | --- | --- | --- |
|  | | |  |  |  |  |  |
| **STUDIES IN GENERAL POPULATION** | | | | | | | |
| BHF-FHS | C-C | UK | Direct media campaign | 4521 | 1998-2003 | 36.67 | 50.53(13.90) |
| BRHS | Cohort | UK | General practice | 3835 | 1998-2000 | 0.00 | 68.74(5.49) |
| BWHHS | Cohort | UK | General practice | 3405 | 1999-2001 | 100.00 | 68.91(5.49) |
| CCHS | Cohort | Denmark | General population | 10375 | 1991-1994 | 55.68 | 56.57(16.26) |
| CYPRUS | Cohort | Cyprus | Mayor’s list | 734 | 2003-2008 | 52.97 | 61.27(10.26) |
| EAS | Cohort | UK | General practices | 857 | 1987 | 50.64 | 64.38(5.82) |
| EPIC-Netherlands | Nested C-C | Netherlands | Existing cohorts | 5194 | 1993-1997 | 78.11 | 54.03(10.22) |
| EPIC-Norfolk | Nested C-C | UK | General practices | 3039 | 1993-1997 | 34.65 | 64.68(7.88) |
| GRAPHIC | Cohort | UK | Nuclear families | 2024 | 2003-2005 | 49.56 | 39.19(14.48) |
| IMPROVE | Cohort | Europe | Clinic | 3236 | 2004-2005 | 52.83 | 64.35(5.17) |
| NPHS-II | Cohort | UK | General practices | 2693 | 1989-1994 | 0.00 | 56.11(3.33) |
| PREVEND | Cohort | Netherlands | Community | 8114 | 1997 | 50.86 | 49.05(12.76) |
| PROCARDIS | C-C | Sweden, UK, Germany, Italy | Hospital | 5463 | 1998-2002 | 41.06 | 60.72(9.04) |
| PROSPER | RCT | UK, Ireland, Netherlands | General practices | 3991 | 1997-1999 | 51.79 | 75.28 (3.35) |
| Rotterdam | Cohort | Netherlands | District participants | 5974 | 1989-1993 | 59.37 | 69.38(8.91) |
| TPT | RCT | UK | General practice | 4014 | 1984-1989 | 0.00 | 56.06(6.74) |
| UCP | Nested C-C | Netherlands | National drug registry | 1632 | 1985-2005 | 25.49 | 62.76 (9.64) |
| UDACS | Cohort | UK | Clinic | 564 | 2001-2002 | 41.13 | 66.73(11.09) |
| Whitehall II | Cohort | UK | Workplace | 5018 | 1985-1988 | 26.44 | 43.90(5.93) |
| **STUDIES IN ACUTE CORONARY SYNDROME PATIENTS** | | | | | | | |
| CURE | RCT | 28 countries | Hospitals | 4334 | 1998-2000 | 40.90 | 64.20(11.00) |
| FAST-MI | Cohort | France | Nationwide ACS registry | 973 | 2005-ongoing | 29.19 | 66.09(13.65) |
| GENDEMIP | Cohort | Czech Republic | 5 Coronary Units | 1432 | 2006-2009 | 25.98 | 57.08(8.62) |
| GRACE-France | Cohort | France | Hospitals | 274 | 2000-2002 | 20.44 | 60.13(12.67) |
| GRACE-Scotland | Cohort | Scotland | Hospital | 1488 | 1999-2009 | 30.04 | 64.84 (12.04) |
| IHCS | Nested C-C | USA | Single hospital | 2382 | 1994-ongoing | 29.14 | 62.99 (12.29) |
| KAROLA | Cohort | Germany | Rehabilitation clinics | 1019 | 1999-2000 | 15.00 | 58.93 (7.96) |
| MERLIN-TIMI 36 | RCT | 17 countries | Hospitals | 1606 | 2004-2007 | 34.10 | 63.43 (10.81) |
| MIRACL | RCT | 19 countries | Hospital | 2587 | 1997-1999 | 34.80 | 65.7 (11.8) |
| PROVE-IT TIMI 22 | RCT | 8 countries | Hospitals | 2260 | 2000-2003 | 22.51 | 57.48 (11.06) |
| **OTHER STUDIES** | | | | | | | |
| AMC-PAS | C-C | Netherlands | Hospital clinic | 740 | 1990-2000 | 19.00 | 44.05(3.91) |
| ASAP | Cohort | Sweden | Hospital clinic | 272 | 2006-2010 | 28.68 | 63.54 (12.39) |
| GENDER | Nested C-C | Netherlands | Hospital clinic | 866 | 1999-2001 | 26.00 | 62.01 (11.09) |
| LIFE Heart | C-C | Germany | Hospital clinic | 3128 | 2006-present | 32.83 | 62.86 (11.52) |
| MedStar | C-C | USA | Single-centre hospital | 1322 | 2004-2007 | 54.59 | 59.76(8.86) |
| PennCath | C-C | USA | Single-centre hospital | 1516 | 1998-2003 | 51.92 | 59.29(9.69) |
| SMART | Cohort | Netherlands | Hospital clinic | 8297 | 1996-1998 | 32.24 | 56.51(12.42) |

**Footnotes** Abbreviations: C-C: case control; ICU: intensive care unit; RCT: randomized clinical trial; X-S: cross sectional. All cohorts were prospective in design. AMC-PAS: Premature Atherosclerosis Patients and Sanquin Blood Bank Controls; ASAP: Advanced Study of Aortic Pathology; BHF-FHS: British Heart Foundation Family Heart Study; BRHS: British Regional Heart Study; BWHHS: British Women’s Health and Heat Study; CCHS: Copenhagen City heart Study; CURE: The Clopidogrel in Unstable angina to prevent Recurrent Events; Cyprus: Cyprus Study; EAS: Edinburgh Artery Study; EPIC: European Prospective Investigation into Cancer and Nutrition; GENDEMIP: GENetic DEterminant of Myocardial Infarction in Prague; GRACE: Global Registry of Acute Coronary Events; GRAPHIC: Genetic Regulation of Arterial Pressure of Humans in the Community; IHCS: Intermountain Heart Collaborative Study; IMPROVE: IMPROVE Study; KAROLA: Langzeiterfolge der KARdiOLogischen Anschlussheilbehandlung; LIFE Heart: Leipzig Heart Study; Med-Star: Med-Star Study; MERLIN-TIMI 36: Metabolic Efficiency With Ranolazine for Less Ischemia in Non−ST-Elevation Acute Coronary Syndromes ; MIRACL: Myocardial Ischemia Reduction with Aggressive Cholesterol Lowering; NPHS-II: Northwick park Heart Study; PennCATH: University of Pennsylvania Catheterization study program; PREVEND: Prevention of Renal and Vascular End-stage Disease; PROCARDIS: PRecOcious Coronary ARtery DISease; PROSPER: PROspective Study of Pravastatin in the Elderly at Risk; PROVE-IT TIMI 22: Pravastatin Or atorVastatin Evaluation and Infection Trial; Rotterdam: Rotterdam Study; SMART: Second Manifestations of ARTerial disease; TPT: Thrombosis Prevention Trial; UDACS: University College London Diabetes And Cardiovascular Disease Study; UCP: Utrecht Cardiovascular Pharmacogenetics; Whitehall II: Whitehall II Study; FAST-MI: French Registry of Acute ST-Elevation or Non–ST-elevation Myocardial Infarction; GRACE: Global Registry of Acute Coronary Events.

## Table 2. Data availability for genotype, sPLA_2_ assays, cardiovascular biomarkers and measures of atherosclerosis in the collaborating studies

|  | **sPLA_2_ assays** | | | **Circulating biomarkers/anthropometric traits** | | | | | | |  | | **Genotype availability** |
| --- | --- | --- | --- | --- | --- | --- | --- | --- | --- | --- | --- | --- | --- |
|  | IIA mass | Enzyme activity | | SBP | LDL-C | HDL-C | TG | IL6 | CRP | Glucose | BMI | C-IMT |
| **STUDIES IN GENERAL POPULATION** | | | | | | | | | | | | | |
| BHF-FHS | - | - | - | | - | - | - | - | - | - | - | - | ● |
| BRHS | - | - | ● | | ● | ● | ● | ● | ● | ● | ● | - | ● |
| BWHHS | - | - | ● | | ● | ● | ● | ● | ● | ● | ● | - | ● |
| CCHS | - | - | ● | | ● | ● | ● | - | ● | ● | ● | - | ● |
| CYPRUS | - | - | ● | | ● | ● | ● | - | ● | ● | ● | ● | ● |
| EAS | - | - | ● | | ● | ● | ● | ● | ● | ● | ● | ● | ● |
| EPIC-Netherlands | - | - | ● | | ● | ● | ● | - | ● | ● | ● | - | ● |
| EPIC-Norfolk | ● | ● | ● | | ● | ● | ● | - | ● | - | ● | - | ● |
| GRAPHIC | - | - | ● | | ● | ● | ● | - | ● | ● | ● | - | ● |
| IMPROVE | - | - | ● | | ● | ● | ● | - | ● | ● | ● | ● | ● |
| NPHS-II | - | - | ● | | ● | ● | ● | - | ● | - | ● | - | ● |
| PREVEND | - | - | ● | | ● | ● | ● | - | ● | ● | ● | ● | ● |
| PROCARDIS | - | - | - | | - | - | - | - | - | - | - | - | ● |
| PROSPER | - | - | ● | | ● | ● | ● | ● | - | - | ● | - | ● |
| Rotterdam | - | - | ● | | - | ● | - | ● | ● | - | ● | ● | ● |
| TPT | - | - | ● | | - | - | - | - | - | - | ● | - | ● |
| UCP | - | - | - | | - | - | - | - | - | - | ● | - | ● |
| UDACS | ● | - | - | | - | - | - | - | - | - | - | - | ● |
| Whitehall II | - | - | ● | | ● | ● | ● | ● | ● | ● | ● | - | ● |
| **STUDIES IN ACUTE CORONARY SYNDROME** | | | | | | | | | | | | | |
| CURE | - | - | - | | - | - | - | - | - | - | - | - | ● |
| FAST-MI | ● | ● | ● | | - | - | - | ● | ● | ● | ● | - | ● |
| GENDEMIP | - | - | ● | | ● | ● | ● | - | ● | ● | ● | - | ● |
| GRACE-France | ● | ● | ● | | ● | - | - | - | ● | - | ● | - | ● |
| GRACE-Scotland | ● | ● | ● | | ● | ● | ● | - | ● | ● | ● | - | ● |
| IHCS | - | - | ● | | ● | ● | ● | - | ● | ● | ● | - | ● |
| KAROLA | ● † | ● † | ● | | ● | ● | ● | ● | ● | ● | ● | - | ● |
| MERLIN-TIMI 36 | - | - | - | | - | - | - | - | - | - | - | - | ● |
| MIRACL | ● | ● | ● | | - | - | - | - | - | - | ● | - | - |
| PROVE-IT TIMI 22 | - | - | - | | - | - | - | - | - | - | - | - | ● |
| **OTHER STUDIES** | | | | | | | | | | | | | |
| AMC-PAS | - | - | ● | | ● | ● | ● | - | ● | ● | ● | - | ● |
| ASAP | - | - | - | | - | - | - | - | - | - | - | - | ● |
| GENDER | - | - | - | | - | - | - | - | - | - | - | - | ● |
| LIFE Heart | - | - | - | | ● | ● | ● | - | ● | ● | ● | ● | ● |
| MedStar | - | - | ● | | ● | ● | - | - | - | - | ● | - | ● |
| PennCath | - | - | ● | | ● | ● | - | - | - | - | ● | - | ● |
| SMART | - | - | - | | - | - | - | - | - | - | - | ● | ● |

**Footnotes** Abbreviations: BMI: body mass index; C-IMT: carotid intima medial thickness; CRP: C-reactive protein; HDL-C: high-density lipoprotein cholesterol; IL6: interleukin 6; LDL-C: low-density lipoprotein cholesterol; SBP: systolic blood pressure; sPLA_2_: secretory phospholipase A2; TG: triglycerides
† not included in the observational or *PLA2G2A*-sPLA_2_ analysis as blood samples were not taken at time of ACS

Table 3. Characteristics of assays in studies conducted to measure sPLA_2_ IIA mass and sPLA_2_ enzyme activity.

| **sPLA_2_ trait** | **Parameter** | **EPIC-Norfolk** | **FAST-MI** | **GRACE-France & Scotland** | **MIRACL** | **UDACS** |
| --- | --- | --- | --- | --- | --- | --- |
| **sPLA_2_-IIA mass** | Assay | ELISA ( Cayman Chemical Company, Ann Arbor, Michigan) | Time-resolved fluoroimmunoassay  (an ELISA-like assay) | ELISA ( Cayman Chemical Company, Ann Arbor, Michigan) | ELISA ( Cayman Chemical Company, Ann Arbor, Michigan) | ELISA ( Cayman Chemical Company, Ann Arbor, Michigan) |
|  | Duplicates | Intra-assay variation between duplicates was 9.2% | Intra and interassay coefficient of variation was <15% | Intra and interassay coefficient of variation was <10% | Intra-assay variation between duplicates was  9.2% | Intra- and inter- assay coefficients of variation were 6.0 and 10.3%, respectively |
|  | Lower detection limit | 0.4 ng/ml | 0.5 ng/ml | 0.02 ng/ml | 0.4 ng/ml | 0.02 ng/ml |
|  | Laboratory | CLB/Sanquin Research lab, Amsterdam | Valbonne, France | Paris Cardiovascular Research Center | Paris Cardiovascular Research Center | Dr Camejo’s lab, Gothenburg University, Sweden |
| **sPLA_2_ enzyme activity** | Assay | Selective fluorometric assay | Selective fluorometric assay | Selective fluorometric assay | Selective fluorometric assay | Not measured |
|  | Duplicates | Intra- and inter-assay coefficient of variation was < 10% | Intra- and inter-assay coefficient of variation was < 10% | Intra and inter-assay coefficient of variation was <10% | Intra and inter-assay coefficient of variation was <10% | N/A |
|  | Lower detection limit | 0.10 nmol/min per ml | 0.10 nmol/min per ml | 0.10 nmol/min/ml | 0.10 nmol/min/ml | N/A |
|  | Laboratory | Paris Cardiovascular Research Center | Paris Cardiovascular Research Center | Paris Cardiovascular Research Center | Paris Cardiovascular Research Center | N/A |

**Footnotes** N/A: not applicable

## Table 4. Genotyping characteristics in the collaborating studies.

|  |  | | ***PLA2G2A* rs11573156 †** | | | | | | **Hardy-Weinberg** | |
| --- | --- | --- | --- | --- | --- | --- | --- | --- | --- | --- |
| **Study** | **Genotyping platform** | | **Call rate (%)** | **Proxy SNP used (r^2^ value for LD)** | **CC** | **CG** | | **GG** | **Estimated disequilibrium coefficient** | **Exact significant probability** |
| **STUDIES IN GENERAL POPULATION** | | | | | | | | | | |
| **BHF-FHS** | | IBC Cardiochip | 100.0 | N/A | 1442 | 839 | | 141 | 0.005 | 0.21 |
| **BRHS** | | KASPar | 97.2 | N/A | 2379 | 1267 | | 186 | 0.003 | 0.31 |
| **BWHHS** | | IBC Cardiochip | 99.8 | N/A | 2066 | 1150 | | 189 | 0.005 | 0.08 |
| **CCHS** | | ABI TaqMan | 99.8 | N/A | 5482 | 4089 | | 804 | 0.002 | 0.28 |
| **CYPRUS** | | ABI TaqMan | 94.3 | N/A | 456 | 236 | | 42 | 0.010 | 0.13 |
| **EAS** | | ABI TaqMan | 96.4 | N/A | 547 | 257 | | 53 | 0.017 | 0.004 |
| **EPIC-Netherlands** | | IBC Cardiochip | 100.0 | N/A | 2961 | 1927 | | 306 | -0.001 | 0.76 |
| **EPIC-Norfolk** | | ABI TaqMan | 91.5 | N/A | 1834 | 1046 | | 159 | 0.002 | 0.53 |
| **GRAPHIC** | | IBC Cardiochip | 100.0 | N/A | 1201 | 728 | | 95 | -0.004 | 0.28 |
| **IMPROVE** | | ABI TaqMan | 91.6 | N/A | 1941 | 1109 | | 186 | 0.005 | 0.10 |
| **NPHS-II** | | ABI TaqMan | 97.0 | N/A | 1617 | 936 | | 140 | 0.001 | 0.78 |
| **PREVEND** | | KASPar | 98.0 | N/A | 4635 | 2974 | | 505 | 0.002 | 0.34 |
| **PROCARDIS** | | IBC Cardiochip | 100.0 | N/A | 1910 | 1216 | | 201 | 0.001 | 0.71 |
| **PROSPER** | | Illumina 660K chip | 97.5 | N/A | 2571 | 1246 | | 174 | 0.004 | 0.15 |
| **Rotterdam** | | Illumina Infinium II HumanHap550 SNP chip array (v3) | 100.0 | Multiple SNPs (0.79) | 3290 | 2322 | | 362 | -0.004 | 0.08 |
| **TPT** | | ABI TaqMan | 97.5 | N/A | 2367 | 1423 | | 224 | 0.001 | 0.60 |
| **UCP** | | IBC Cardio | 100.0 | N/A | 939 | 595 | | 98 | 0.001 | 0.79 |
| **UDACS** | | ABI TaqMan | 94.2 | N/A | 337 | 195 | | 32 | 0.004 | 0.63 |
| **Whitehall II** | | IBC Cardio | 91.7 | N/A | 2992 | 1774 | | 252 | -0.001 | 0.63 |
| **STUDIES IN ACUTE CORONARY SYNDROME** | | | | | | | | | | |
| **CURE** | | ABI TaqMan | 98.9 | N/A | 2475 | 1578 | 281 | | 0.0039 | 0.18 |
| **FAST-MI** | | SNPlex | 98.3 | N/A | 623 | 301 | 49 | | 0.008 | 0.12 |
| **GENDEMIP** | | PCR-RFLP | 96.7 | N/A | 750 | 559 | 123 | | 0.007 | 0.19 |
| **GRACE France** | | ABI TaqMan | 97.8 | N/A | 178 | 87 | 9 | | -0.004 | 0.85 |
| **GRACE Scotland** | | ABI TaqMan | 97.8 | N/A | 901 | 506 | 81 | | 0.004 | 0.37 |
| **IHCS** | | ABI TaqMan | 92.4 | N/A | 1475 | 792 | 115 | | 0.002 | 0.50 |
| **KAROLA** | | ABI TaqMan | 98.9 | N/A | 560 | 399 | 49 | | -0.012 | 0.04 |
| **MERLIN-TIMI 36** | | IBC Cardio | 99.9 | N/A | 935 | 578 | 93 | | 0.001 | 0.78 |
| **PROVE-IT TIMI 22** | | IBC Cardio | 99.7 | N/A | 1392 | 756 | 112 | | 0.003 | 0.50 |
| **OTHER STUDIES** | | | | | | | | | | |
| **AMC-PAS** | | IBC Cardiochip | 99.6 | N/A | 117 | 64 | 10 | | 0.004 | 0.83 |
| **GENDER** | | Illumina Human 610-Quad | 100.0 | rs10732279 (0.91) | 328 | 211 | 32 | | -0.002 | 0.91 |
| **LIFE Heart** | | Homogeneous fluorescence-based melting curve† | 95.5 | N/A | 1278 | 902 | 135 | | -0.006 | 0.15 |
| **MedStar** | | Affymetrix 6.0 | 98.3 | Multiple SNPs (0.77) | 726 | 511 | 85 | | -0.006 | 0.62 |
| **PennCath** | | IBC Cardio | 98.6 | N/A | 916 | 540 | 60 | | -0.017 | 0.03 |
| **SMART** | | KASPar | 98.2 | N/A | 4649 | 2882 | 465 | | 0.001 | 0.52 |

**Footnotes** Abbreviations: LD: linkage disequilibrium; MVE: major vascular event; N/A (not applicable) in column titled. “Proxy SNP used” indicates rs11573156 directly genotyped; †methodology described in Holdt et al † For case control studies, the genotype values are limited to controls.

## Table 5. Contribution of each study to individual outcomes and primary outcome for the Mendelian randomization analysis.

| **Study** | **Prevalent** | | | **Incident/Recurrent** | | | | | **Contributes to primary outcome (MVE)** |
| --- | --- | --- | --- | --- | --- | --- | --- | --- | --- |
|  | **MI** | **Stroke** | **Coronary Stenosis** | **Non-fatal MI** | **Non-fatal Stroke** | | **Fatal MI or stroke** | **All-cause mortality** |  |
| **STUDIES IN GENERAL POPULATION** | | | | | | | | | |
| **BHF-FHS** | ● | - | - | - | - | | - | - | ● |
| **BRHS** | ● | ● | - | ● | ● | | ● | - | ● |
| **BWHHS** | ● | ● | - | ● | ● | | ● | - | ● |
| **CCHS** | ● | ● | - | ● | ● | | ● | - | ● |
| **CYPRUS** | ● | ● | - | - | - | | - | - | ● |
| **EAS** | ● | ● | - | ● | ● | | ● | - | ● |
| **EPIC-Netherlands** | - | - | - | ● | ● | | ● | - | ● |
| **EPIC-Norfolk** | - | - | - | ● | - | | ● | - | ● |
| **GRAPHIC** | - | - | - | - | - | | - | - | - |
| **IMPROVE** | ● | - | - | ● | ● | | ● | - | ● |
| **NPHS-II** | - | - | - | ● | ● | | ● | - | ● |
| **PREVEND** | ● | ● | - | ● | ● | | ● | - | ● |
| **PROCARDIS** | ● | - | - | - | - | | - | - | ● |
| **PROSPER** | ● | ● | - | ● | ● | | ● | - | ● |
| **Rotterdam** | ● | ● | - | ● | ● | | ● | - | ● |
| **TPT** | - | - | - | ● | ● | | ● | - | ● |
| **UCP** | - | - | - | ● | - | | - | - | ● |
| **UDACS** | - | - | - | - | - | | - | - | - |
| **Whitehall II** | ● | - | - | - | - | | - | - | ● |
| **STUDIES IN ACUTE CORONARY SYNDROME** | | | | | | | | | |
| **CURE** | - | - | - | ● | ● | ● | | - | ● |
| **FAST-MI** | - | - | - | ● | ● | - | | ● | ● |
| **GENDEMIP** | - | - | - | - | - | ● | | - | ● |
| **GRACE France** | - | - | - | ● | - | - | | ● | ● |
| **GRACE Scotland** | - | - | - | ● | ● | - | | ● | ● |
| **IHCS** | - | - | - | ● | ● | ● | | - | ● |
| **KAROLA** | - | - | - | ● | ● | ● | | - | ● |
| **MERLIN-TIMI 36** | - | - | - | ● | - | ● | | - | ● |
| **MIRACL** | - | - | - | - | - | - | | - | - |
| **PROVE-IT TIMI 22** | - | - | - | ● | ● | ● | | - | ● |
| **OTHER STUDIES** | | | | | | | | | |
| **AMC-PAS** | ● | - | ● | - | - | - | | - | - |
| **GENDER** | - | - | - | - | - | - | | - | - |
| **LIFE Heart** | ● | - | ● | - | - | - | | - | - |
| **MedStar** | - | - | ● | - | - | - | | - | - |
| **PennCath** | ● | - | ● | - | - | - | | - | - |
| **SMART** | - | - | - | ● | ● | ● | | - | - |

## Table 6. Assessment of departure from linearity in the relationship between sPLA_2_-IIA mass, sPLA_2_ enzyme activity and cardiovascular events, adjusted for age and gender.

| **Study/Setting** | **Event** | **One log unit reduction in**  **sPLA_2_-IIA mass** | | | **One log unit reduction in**  **sPLA_2_ enzyme activity** | | |
| --- | --- | --- | --- | --- | --- | --- | --- |
|  |  | **N** | **Linear OR (95%CI)** | **LRT P-value** | **N** | **Linear OR (95%CI)** | **LRT P-value** |
| **General population** |  |  |  |  |  |  |  |
| EPIC-Norfolk | Fatal/nonfatal MI | 3371 | 0.67 (0.59, 0.75) | 0.747 | 3321 | 0.30 (0.22, 0.41) | 0.910 |
| **Acute coronary syndrome** | |  |  |  |  |  |  |
| GRACE-Scotland | Death/MI | 158 | 0.77 (0.49, 1.19) | 0.737 | 164 | 0.46 (0.14, 1.48) | 0.100 |
| GRACE-France | Death/MI | 277 | 0.95 (0.64, 1.42) | 0.195 | 278 | 0.40 (0.16, 1.00) | 0.165 |
| FAST-MI | Death/MI | 855 | 1.14 (0.87,1.49) | 0.642 | 1011 | 0.68 (0.40, 1.14) | 0.165 |

**Footnotes** A logistic regression model was fitted with fatal/nonfatal MI (general population cohort) or nonfatal MI/all-cause mortality (ACS cohorts) as the dependent variable and sPLA_2_-IIA mass or log sPLA_2_ enzyme activity as a continuous independent variable, assuming a linear relationship between log sPLA_2_ and cardiovascular events. A quadratic term (for each of sPLA_2_-IIA mass and sPLA_2_ enzyme activity) was then fitted in addition to the linear covariate. The Likelihood Ratio Test was used to test the null hypothesis, that the simpler model (without the quadratic term) better represented the data. The large P values derived from LRT provide no evidence for a quadratic relationship, which argues in favour of a log-linear relationship between sPLA_2_-IIA mass, sPLA_2_ enzyme activity and cardiovascular events.

## Table 7. Association between tertiles of sPLA_2_-IIA mass and traditional cardiovascular risk factors in 3371 individuals in EPIC-Norfolk.

|  | **Tertile (min-max, ng/ml) of sPLA_2_-IIA mass** | | | | | | | | |  |
| --- | --- | --- | --- | --- | --- | --- | --- | --- | --- | --- |
|  | **1 (0.4-6.8)** | | | **2 (6.8-11.3)** | | | **3 (11.3-114.5)** | | |  |
| **Continuous traits** | **N** | **Mean** | **SD** | **N** | **Mean** | **SD** | **N** | **Mean** | **SD** | **P value §** |
| **Age (yrs)** | 1125 | 63.51 | 8.24 | 1124 | 64.93 | 7.59 | 1122 | 66.07 | 7.17 | 1.10x10^-17^ |
| **BMI (kg/m^2^)** | 1125 | 26.20 | 3.28 | 1120 | 26.69 | 3.66 | 1122 | 27.03 | 3.99 | 5.46x10^-9^ |
| **CRP* (mg/dl)** | 1100 | 0.22 | 1.05 | 1108 | 0.53 | 1.15 | 1104 | 1.01 | 1.21 | 1.67x10^-74^ |
| **Systolic BP (mmHg)** | 1122 | 139.54 | 17.85 | 1122 | 140.63 | 17.64 | 1120 | 141.83 | 19.21 | 4.36x10^-3^ |
| **Diastolic BP (mmHg)** | 1122 | 84.13 | 11.08 | 1122 | 84.49 | 11.20 | 1120 | 84.51 | 11.99 | 0.69 |
| **HDL-C (mmol/l)** | 1073 | 1.31 | 0.38 | 1051 | 1.35 | 0.40 | 1059 | 1.36 | 0.40 | 0.03 |
| **LDL-C (mmol/l)** | 1073 | 4.05 | 0.98 | 1052 | 4.17 | 1.03 | 1059 | 4.20 | 1.05 | 0.01 |
| **Triglyceride* (mmol/l)** | 1114 | 0.57 | 0.50 | 1106 | 0.56 | 0.51 | 1105 | 0.55 | 0.51 | 0.06 |
| **Apolipoprotein A1 (mg/dl)** | 977 | 156.23 | 28.26 | 972 | 160.14 | 28.58 | 972 | 163.27 | 31.02 | 9.03x10^-6^ |
| **Apolipoprotein B (mg/dl)** | 1029 | 129.79 | 32.28 | 1024 | 133.38 | 31.28 | 1038 | 134.31 | 33.42 | 0.07 |
| **Binary traits** | **N** | **Proportion** | **SD** | **N** | **Proportion** | **SD** | **N** | **Proportion** | **SD** | **P value** ᶲ |
| **Female sex (%)** | 1125 | 21.33 | 40.98 | 1124 | 35.68 | 47.93 | 1122 | 53.21 | 49.92 | 8.94x10^-59^ |
| **T2D (%)** | 1124 | 3.20 | 17.62 | 1123 | 3.03 | 17.14 | 1120 | 4.11 | 19.85 | 0.06 |
| **Ever smoker (%)** | 1116 | 62.01 | 48.56 | 1106 | 62.30 | 48.49 | 1113 | 62.80 | 48.35 | 0.78 |

**Footnotes** Abbreviations: BMI: body mass index; CRP: C-reactive protein; HDL-C: high density lipoprotein cholesterol; LDL-C: low density lipoprotein cholesterol; T2D: type 2 diabetes
* log-transformed (units do not apply); § derived from univariate linear regression using log sPLA2-IIA as the independent variable; ᶲ derived from univariate logistic regression using log sPLA_2_-IIA mass as the independent variable.

Table 8. Association between tertiles of sPLA_2_ enzyme activity and traditional cardiovascular risk factors in 3371 individuals in EPIC-Norfolk**.**

| **Tertile (min-max, nmol/min/ml) of sPLA_2_ enzyme activity** | | | | | | | | | | |
| --- | --- | --- | --- | --- | --- | --- | --- | --- | --- | --- |
|  | **1 (1.7-4.0)** | | | **2 (4.0-4.9)** | | | **3 (4.9-23.1)** | | |  |
| **Continuous traits** | **N** | **Mean** | **SD** | **N** | **Mean** | **SD** | **N** | **Mean** | **SD** | **P value §** |
| **Age (yrs)** | 1107 | 64.19 | 7.84 | 1107 | 64.54 | 7.89 | 1107 | 65.80 | 7.48 | 7.17x10^-7^ |
| **BMI (kg/m^2^)** | 1107 | 26.02 | 3.44 | 1106 | 26.61 | 3.75 | 1104 | 27.32 | 3.71 | 1.00x10^-15^ |
| **CRP* (mg/dl)** | 1086 | 0.41 | 1.20 | 1087 | 0.53 | 1.16 | 1090 | 0.83 | 1.14 | 1.87x10^-16^ |
| **Systolic BP (mmHg)** | 1103 | 138.62 | 18.33 | 1105 | 139.76 | 18.13 | 1106 | 143.71 | 18.01 | 7.46x10^-10^ |
| **Diastolic BP (mmHg)** | 1103 | 83.22 | 11.34 | 1105 | 83.88 | 11.02 | 1106 | 86.03 | 11.69 | 2.57x10^-7^ |
| **HDL-C (mmol/l)** | 1088 | 1.38 | 0.4 | 1070 | 1.35 | 0.4 | 980 | 1.28 | 0.38 | 2.99x10^-8^ |
| **LDL-C (mmol/l)** | 1088 | 3.93 | 0.94 | 1070 | 4.14 | 0.99 | 981 | 4.37 | 1.10 | 4.26x10^-21^ |
| **Triglyceride* (mmol/l)** | 1091 | 0.29 | 0.39 | 1093 | 0.52 | 0.44 | 1092 | 0.87 | 0.50 | 5.40x10^-168^ |
| **Apolipoprotein A1 (mg/dl)** | 976 | 159.74 | 28.98 | 956 | 160.62 | 29.45 | 955 | 159.18 | 29.93 | 0.94 |
| **Apolipoprotein B (mg/dl)** | 1038 | 120.49 | 26.91 | 1024 | 130.77 | 29.12 | 993 | 146.7 | 35.2 | 5.61x10^-72^ |
| **Binary traits** | **N** | **Proportion** | **SD** | **N** | **Proportion** | **SD** | **N** | **Proportion** | **SD** | **P value** ᶲ |
| **Female sex (%)** | 1107 | 33.06 | 47.07 | 1107 | 35.95 | 48.01 | 1107 | 41.73 | 49.33 | 1.27x10^-5^ |
| **T2D (%)** | 1107 | 2.62 | 15.98 | 1105 | 4.43 | 20.60 | 1106 | 3.35 | 17.99 | 0.10 |
| **Ever smoker (%)** | 1095 | 60.00 | 49.01 | 1098 | 59.56 | 49.10 | 1092 | 67.58 | 46.83 | 2.42x10^-4^ |

**Footnotes** Abbreviations: BMI: body mass index; CRP: C-reactive protein; HDL-C: high density lipoprotein cholesterol; LDL-C: low density lipoprotein cholesterol; T2D: type 2 diabetes
* log-transformed (units do not apply); § derived from univariate linear regression using log sPLA_2_ enzyme activity as the independent variable; ᶲ derived from univariate logistic regression using log sPLA_2_ enzyme activity as the independent variable.

## Table 9. Summary associations of *PLA2G2A* rs11573156 per C allele with cardiovascular risk factors and markers of atherosclerosis in studies set in the general population.

| **Biomarker (units)** | **No. of studies (individuals)** | **Summary effect**  **(95% CI)** | **P value**  **(I^2^, %)** |
| --- | --- | --- | --- |
| Systolic BP (mmHg) | 18 (63,325) | -0.11 (-0.36, 0.14) | 0.40 (24) |
| Diastolic BP (mmHg) | 18 (63,326) | -0.10 (-0.24, 0.04) | 0.17 (30) |
| Glucose (mmol/l) | 12 (43,186) | 0.01 (-0.01, 0.03) | 0.32 (12) |
| BMI (kg/m^2^) | 20 (67,764) | 0.02 (-0.03, 0.07) | 0.39 (18) |
| CIMT (mm)* | 6 (12,237) | -0.34 (-0.91, 0.24) | 0.25 (0) |

**Footnotes** Abbreviations: BMI: body mass index; CIMT: carotid intima medial thickness; DBP: diastolic blood pressure; SBP: systolic blood pressure.

* percentage difference as variable log transformed (units do not apply).

## Table 10. Summary effects of *PLA2G2A* rs11573156 per C allele with potential confounders in studies set in the general population.

| **Trait** | **No. of studies**  **(cases/non-cases)** | **Odds Ratio (95% CI)** | **P value (I^2^, %)** |
| --- | --- | --- | --- |
| Age (yrs) | 20 (68,387) † | -0.04 (-0.12, 0.05)† | 0.36 (15) |
| Gender (female/all) | 14 (28,578/57,861) | 1.00 (0.98, 1.03) | 0.75 (0) |
| Smoking status (ever smoker/never smoker) | 19 (39,790/69,614) | 1.00 (0.98, 1.03) | 0.78 (6) |
| T2D (prevalent) | 18 (5,773/63,097) | 1.05 (1.00, 1.10) | 0.06 (16) |
| Statin treatment | 13 (8,284/45,509) | 1.00 (0.95, 1.05) | 0.93 (28) |
| BP-lowering treatment | 13 (10,810/48,738) | 1.02 (0.98, 1.06) | 0.31 (9) |

**Footnotes** Abbreviations: T2D: type 2 diabetes. † effect estimate represents beta coefficient for age, with number in brackets representing the total number of participants.

## Table 11. Summary effects of varespladib (500mg/day) and *PLA2G2A* rs11573156 on biomarkers reported in randomized trials.

|  | **Randomized trials of varespladib (500mg/day)** | | | **Genetic studies of rs11573156 (per C allele)** | | |
| --- | --- | --- | --- | --- | --- | --- |
| **Biomarker (units)** | **No. of trials (individuals)** | **Summary effect (95% CI) *** | **P value,**  **(*I*^2^, %)** | **No. of studies (individuals)** | **Summary effect**  **(95% CI)** | **P value**  **(*I*^2^, %)** |
| **sPLA_2_ MEASURES** |  |  |  |  |  |  |
| sPLA_2_ IIA mass (pmol/L)† | 3 (624) | -13.13 (-21.99, -4.27) | 4 x 10^-3^ (0) | 1 (3035) | -38.62 (-40.49, -36.68) | 2.61x10^-204^ (-) |
| sPLA_2_ enzyme activity (nmol/min/L)† | - | - | - | 1 (2990) | -2.96 (-4.47, -1.42) | 1.8x10^-4^ (-) |
| **REPORTED IN RCT WITH >500 INDIVIDUALS** | | | |  |  |  |
| HDL-C (mmol/l) | 3 (637) | 0.01 (-0.06, 0.07) | 0.84 (0) | 18 (59,086) | 0.000 (-0.005, 0.005) | 0.96 (0) |
| LDL-C (mmol/l) | 3 (610) | -0.20 (-0.42, 0.01) | 0.063 (0) | 17 (53,465) | 0.01 (-0.01, 0.02) | 0.40 (42) |
| Triglyceride (mmol/l)† | 3 (637) | -0.12 (-0.35, 0.11) | 0.32 (0) | 15 (53,079) | 0.78 (0.04, 1.50) | 0.04 (12) |
| Interleukin-6 (pmol/L)† | 1 (513) | 0.00 (-6.34, 6.34) | 1 (0) | 6 (14,980) | 2.54 (0.68, 4.44) | 0.01 (0) |
| **EXPLORATORY BIOMARKERS** | | | |  |  |  |
| C-reactive Protein (mg/l)† | 2 (467) | -0.32 (-3.50, 2.86) | 0.84 (0) | 15 (52,348) | 0.71 (-0.68. 2.12) | 0.32 (0) |
| Fibrinogen (g/l)† | - | - | - | 11 (35,430) | 0.005 (0.001, 0.009) | 0.02 (0) |
| Apolipoprotein A (g/l) | - | - | - | 7 (27,012) | 0.001 (-0.005, 0.007) | 0.66 (0) |
| Apolipoprotein B †ǂ | 2 (249) | -0.78 (-1.05, -0.52) | 8.0x10^-9^ (94) | 7 (27,168) | 0.43 (-0.11, 0.98) | 0.12 (26) |
| LDL size (nm) | 2 (247) | 0.23 (0.19, 0.26) | 1.4x10^-41^ (19) | 1 (2774) | 0.01 (-0.03, 0.05) | 0.57 (-) |
| HDL size (µm) | - | - | - | 1 (2774) | 0.01 (-0.02, 0.04) | 0.60 (-) |
| LDL particles (nmol/l) | 2 (247) | -19.01 (-516.86, 478.84) | 0.94 (0) | 1 (2774) | 3.70 (-23.19, 30.60) | 0.79 (-) |
| HDL particles (umol/l) | 1 (157) | 0.70 (-8.08, 9.48) | 0.88 (-) | 1 (2774) | -0.16 (-0.51, 0.19) | 0.38 (-) |

**Footnotes** Abbreviations: LDL-C: low density lipoprotein cholesterol; HDL-C: high density lipoprotein cholesterol; sPLA_2_: secretory phospholipase A_2_. † percentage difference presented for the genetic analysis as variable log transformed (units do not apply) * represents mean difference in biomarker from baseline to 8 weeks (most widely-reported time-point in randomized trials) comparing varespladib 500mg/day to placebo ǂ RCT summary effect for Apolipoprotein B is the standardized mean difference as different units reported in RCTs.

## Table 12. Characteristics of the randomized trials of varespladib identified in the systematic review

| **Trial name** | **RCT phase** | **Years conducted** | **Clinical setting** | **Primary end-point** | **Age,**  **mean (SD)** | **Sex,**  **% female** | **Number randomised** | **Varespladib dose,**  **mg (dosing frequency per day)** | **Trial duration, months** |
| --- | --- | --- | --- | --- | --- | --- | --- | --- | --- |
| **FRANCIS-ACS** | IIB | 2008-2010 | ACS | Change in LDL-C from baseline to 8 weeks | 59.05 (10.5) | 25.3 | 625 | 500  (once) | 6 |
| **PLASMA** | II | 2007 | Stable CHD | Change in sPLA_2_ IIA concentration or sPLA_2_ enzyme activity from baseline to 8 weeks | 62 (11) | 24 | 393 | 50/100/250/500  (twice) | 2 |
| **PLASMA 2** | II | 2007 | Stable CHD | Change in sPLA_2_ concentration from baseline to 8 weeks | 64 (12) | 11 | 138 | 250/500  (once) | 2 |
| **SPIDER-PCI** | II | 2007-2009 | Stable CHD patients undergoing elective PCI | Elevation of cardiac enzymes (CK-MB, Troponin I) up to 24h after PCI | 63.4 (9.9) | 12.5 | 144 | 500  (twice) | 1 |

**Footnotes** Abbreviations: ACS: acute coronary syndrome; sPLA_2_: secretory phospholipase A_2_, CK-MB: creatine kinase MB isoform; LDL-C: low density lipoprotein cholesterol.

## Table 13. Meta-analysis pooled estimates of the per C allele association between *PLA2G2A* rs11573156 and major vascular events (including individual components) stratified by clinical setting using random effects modelling.

| **Outcome** | **Studies (Events/Total)** | **Odds Ratio (95%CI)** | ***I*^2^,% (95%CI)** |
| --- | --- | --- | --- |
| **General Population: Incident** | | | |
| Major vascular events | 13 (8021/56359) | 1.03 (0.98, 1.08) | 26 (0, 51) |
| Nonfatal MI | 13 (4208/51016) | 1.05 (0.98, 1.13) | 23 (0, 60) |
| Nonfatal Stroke | 11 (2304/46790) | 1.00 (0.92, 1.09) | 19 (0, 59) |
| Fatal MI/Stroke | 12 (1509/48118) | 1.02 (0.90, 1.15) | 41 (0, 70) |
| **General Population: Prevalent** | | | |
| Major vascular events | 12 (7513/55523) | 1.00 (0.94, 1.06) | 38 (0, 64) |
| MI | 12 (6411/54884) | 0.98 (0.91, 1.07) | 52 (8, 75) |
| Stroke | 8 (1102/37280) | 1.03 (0.93, 1.15) | 0 (0, 68) |
| **Acute Coronary Syndrome: Recurrent** | | | |
| Major vascular events | 9 (2520/15768) | 0.96 (0.90, 1.03) | 0 (0, 45) |
| Nonfatal MI | 8 (1158/14152) | 0.97 (0.85, 1.10) | 28 (0, 68) |
| Nonfatal Stroke | 6 (223/12283) | 0.85 (0.69, 1.06) | 0 (0, 75) |
| Fatal MI/Stroke † | 9 (1139/15724) | 0.96 (0.87, 1.06) | 0 (0, 65) |

**Footnotes** † Fatal MI/stroke includes death for some ACS studies (see **eTable 5** for further details).


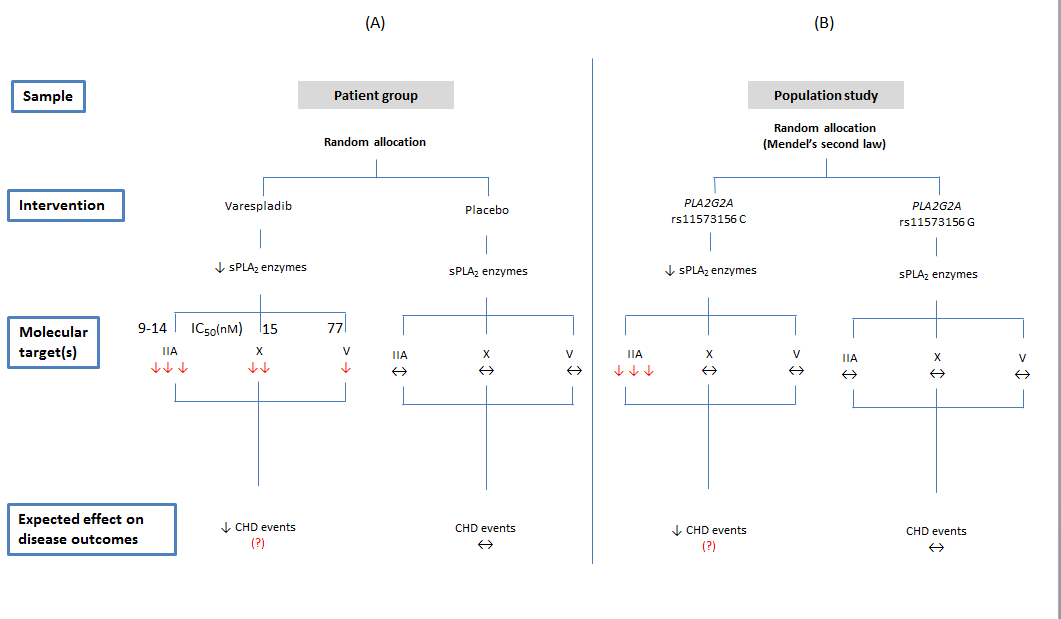
Figure 1. Comparison of (A) a randomized trial of varespladib with (B) random allocation of *PLA2G2A* rs11573156**.**

**Footnotes** Varespladib has greatest effect on sPLA_2_-IIA but additional effects on sPLA_2_-V and X, whereas *PLA2G2A* rs11573156 is specific for sPLA_2_-IIA.

##
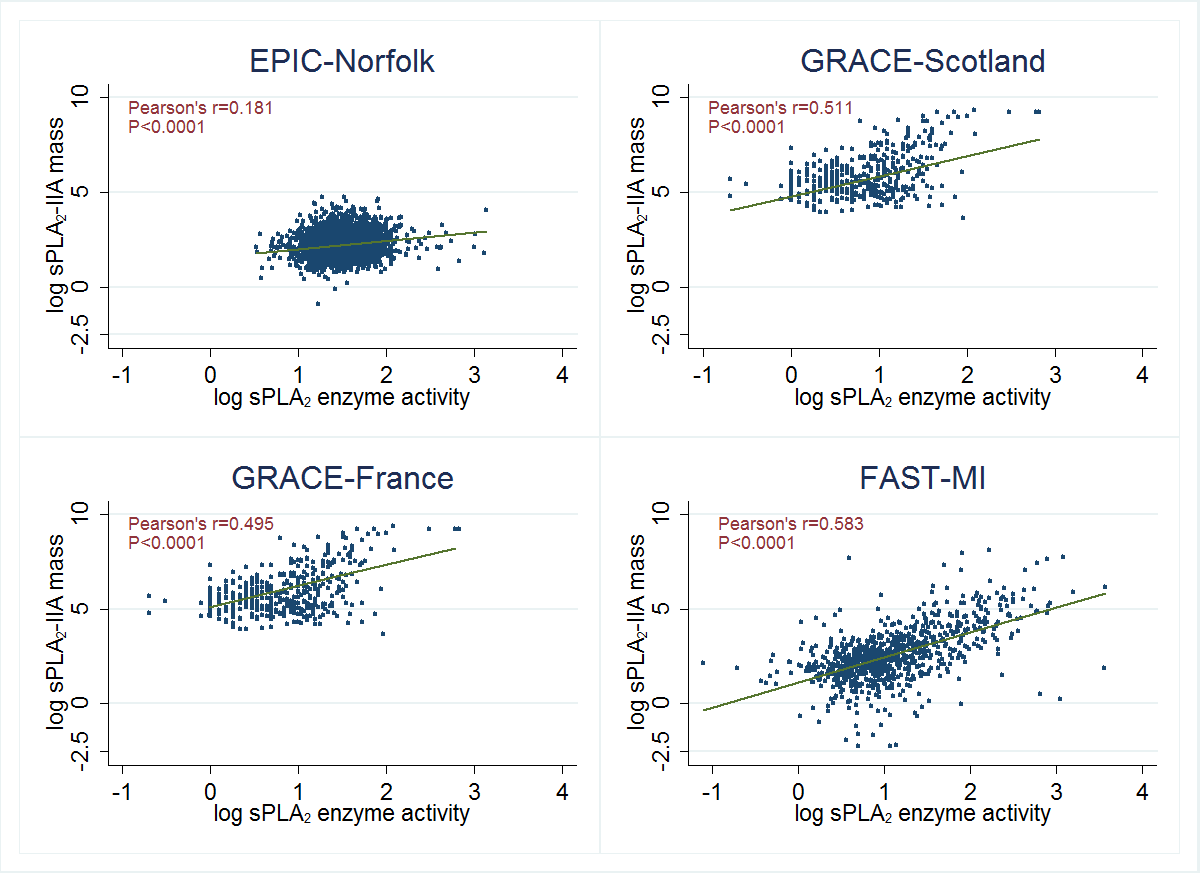
Figure 2. Correlation between sPLA_2_-IIA mass and sPLA_2_ enzyme activity in studies with both measures

##
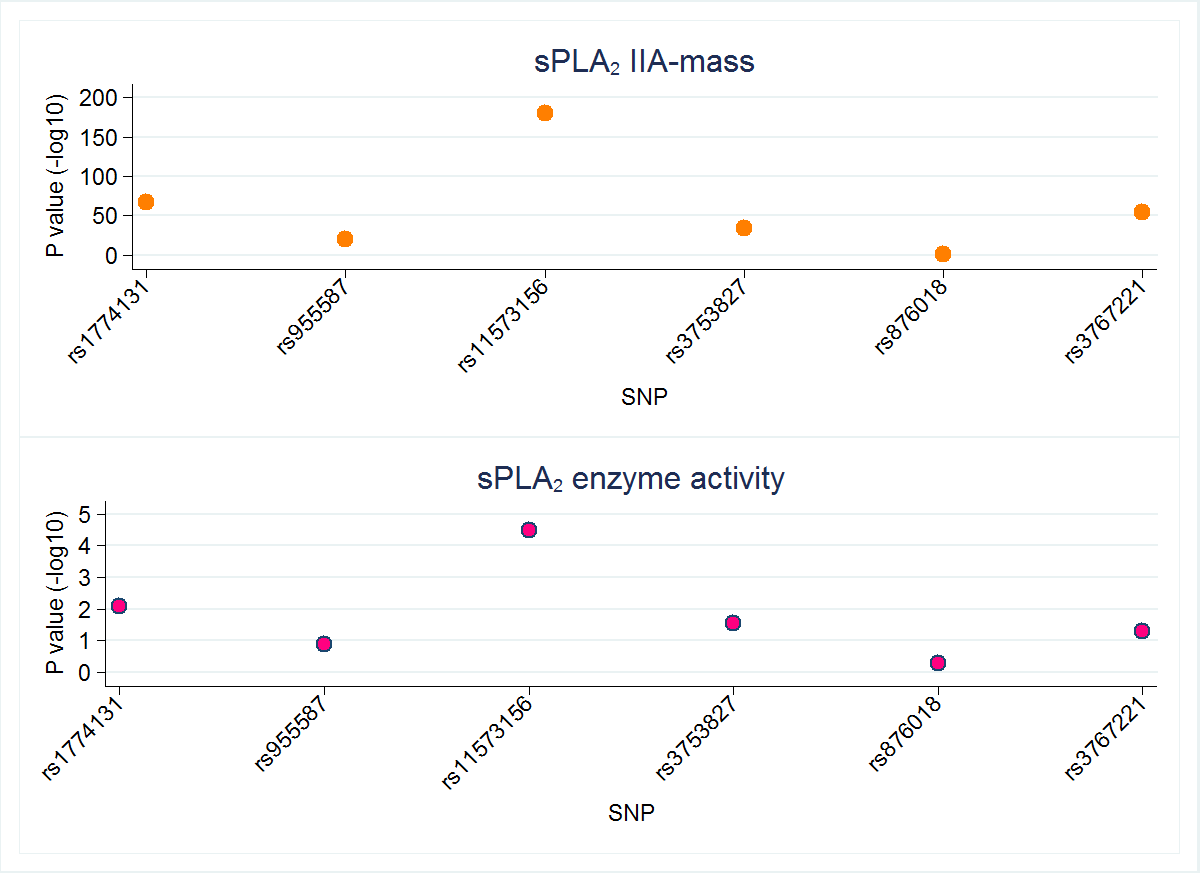
Figure 3. P-values for the associations between the 6 tagging SNPs and sPLA_2_-IIA mass and sPLA_2_ enzyme activity.

**
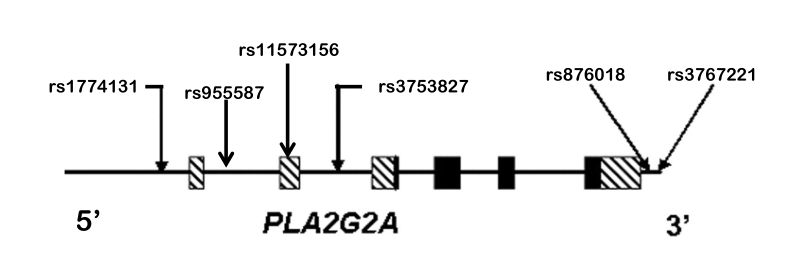
**

**Footnotes** P values represent values from combined analysis in EPIC-Norfolk, GRACE-France and UDACS. In the lower figure, the fully shaded boxes represent coding (translated) exons whereas the hatched boxes are non-coding exons (un-translated).

## Figure 4. Genotype frequency of *PLA2G2A* rs11573156 in the 34 studies.

**
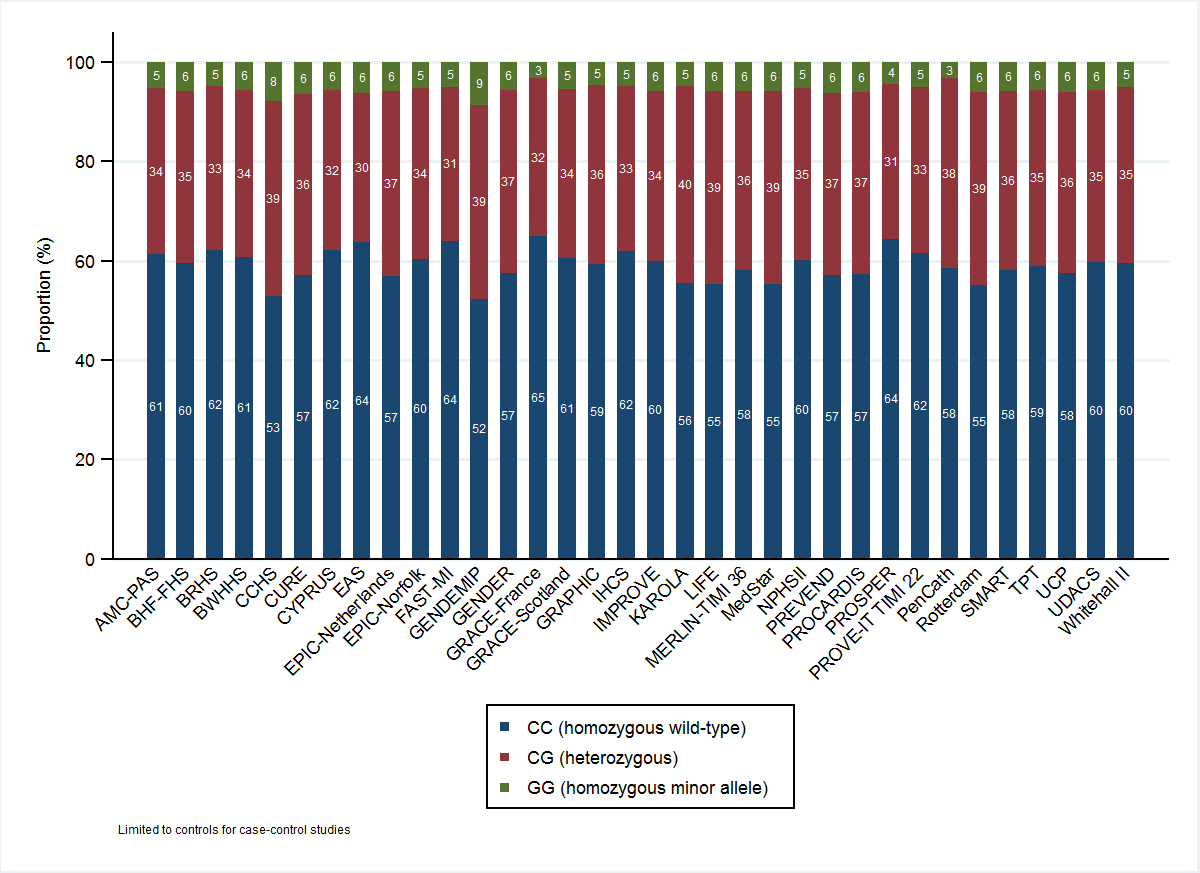
**

**Footnotes:** limited to controls for case-control studies (see **eTable 1**); genotype frequencies are not plotted for ASAP (which doesn’t contribute towards the Mendelian randomization analysis) or for MIRACL (in which genotype information was not available, see **eTable 2**)

##
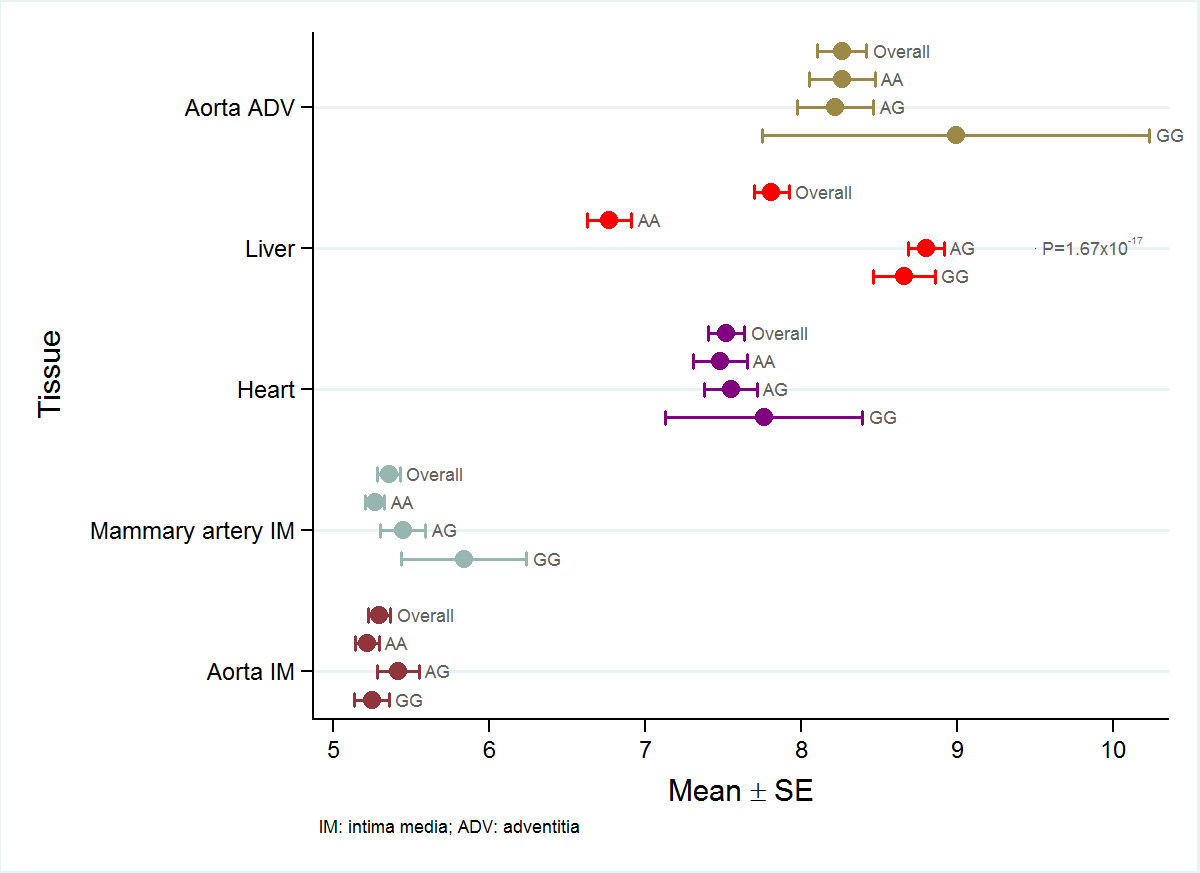
Figure 5. Differential expression of *PLA2G2A* mRNA by rs10732279 genotype. P-value corresponds to the differential expression by genotype.

## Figure 6. PRISMA flow diagram illustrating the search strategy used to identify randomized trials of sPLA_2_ lowering therapies.

**
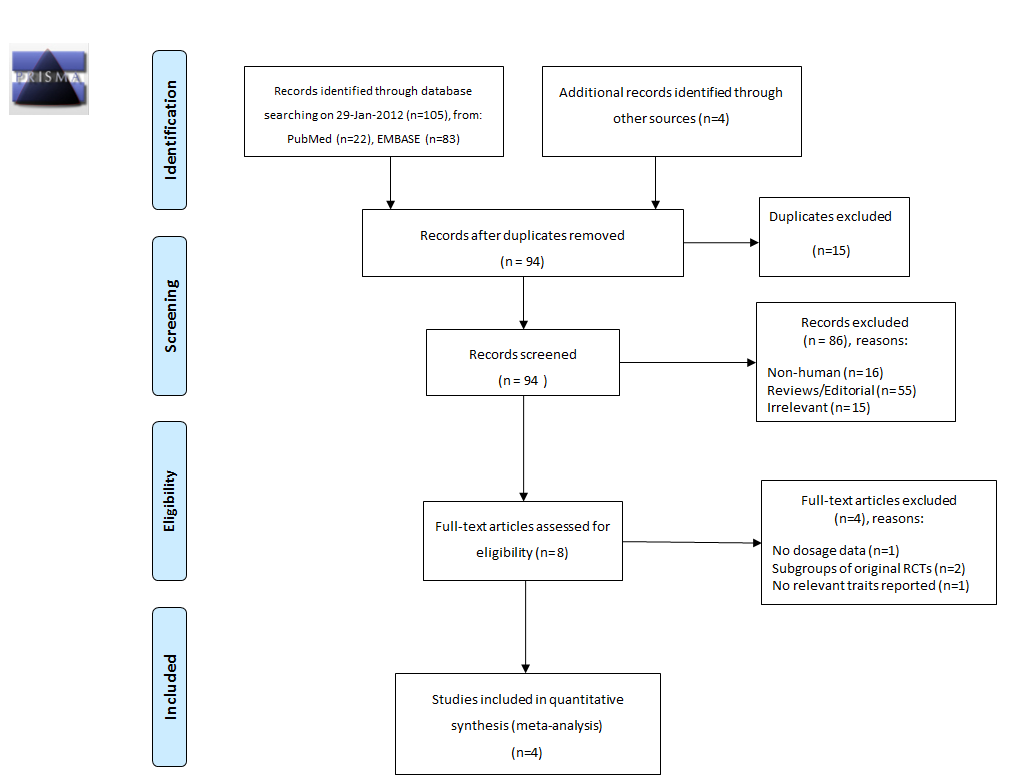
**

## Figure 7. Dose-response relationship between varespladib and sPLA_2_-IIA mass reported in randomized trials.

**
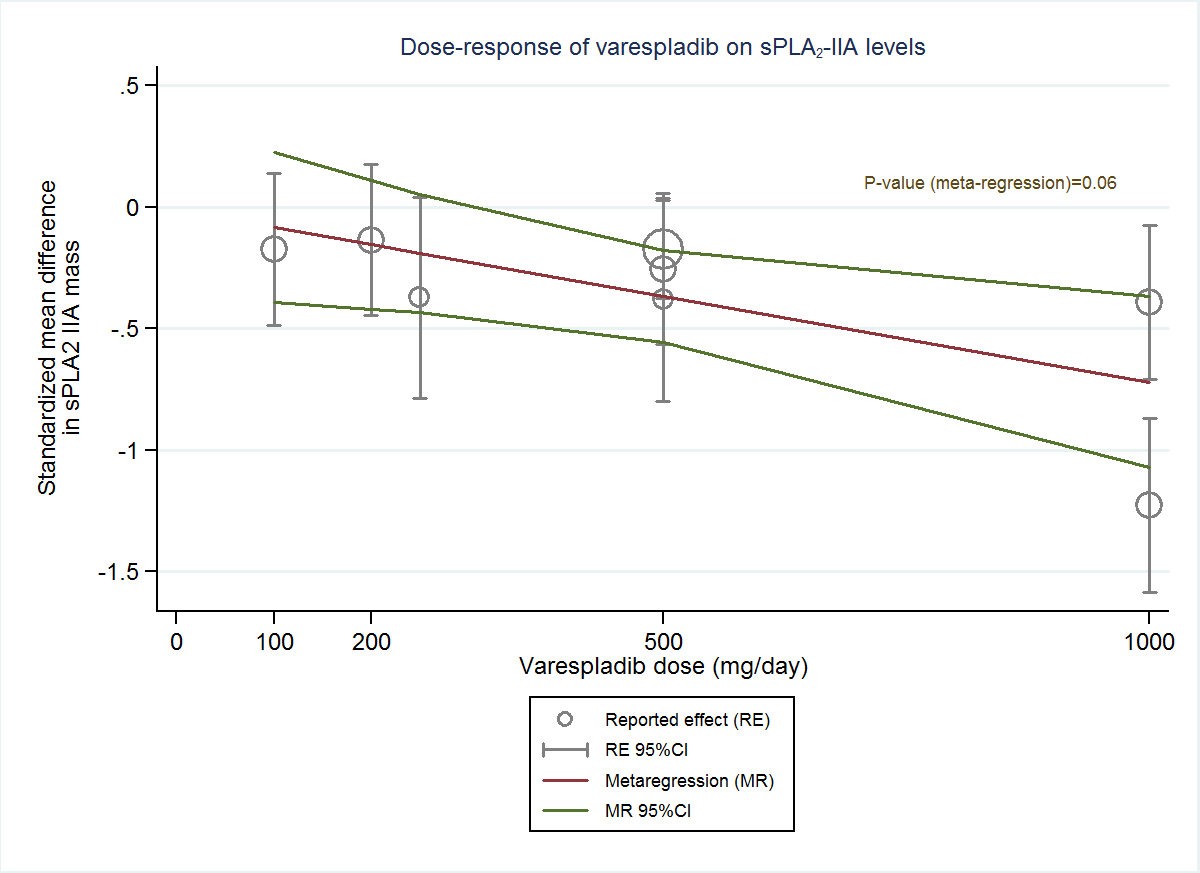
**

**Footnotes** Grey circles and vertical whiskers represent mean (±95%CI) study-level difference in sPLA_2_-IIA mass from baseline to 8 weeks comparing varespladib to placebo. Red line represents meta-regression slope (±95%CI, green lines) of dose of varespladib and difference in sPLA_2_-IIA mass.

## Figure 8. Meta-analysis of the effect of *PLA2G2A* rs11573156 in general population studies and risk of: (a) incident nonfatal myocardial infarction (b) incident nonfatal stroke; (c) fatal myocardial infarction or stroke.

**(a)**
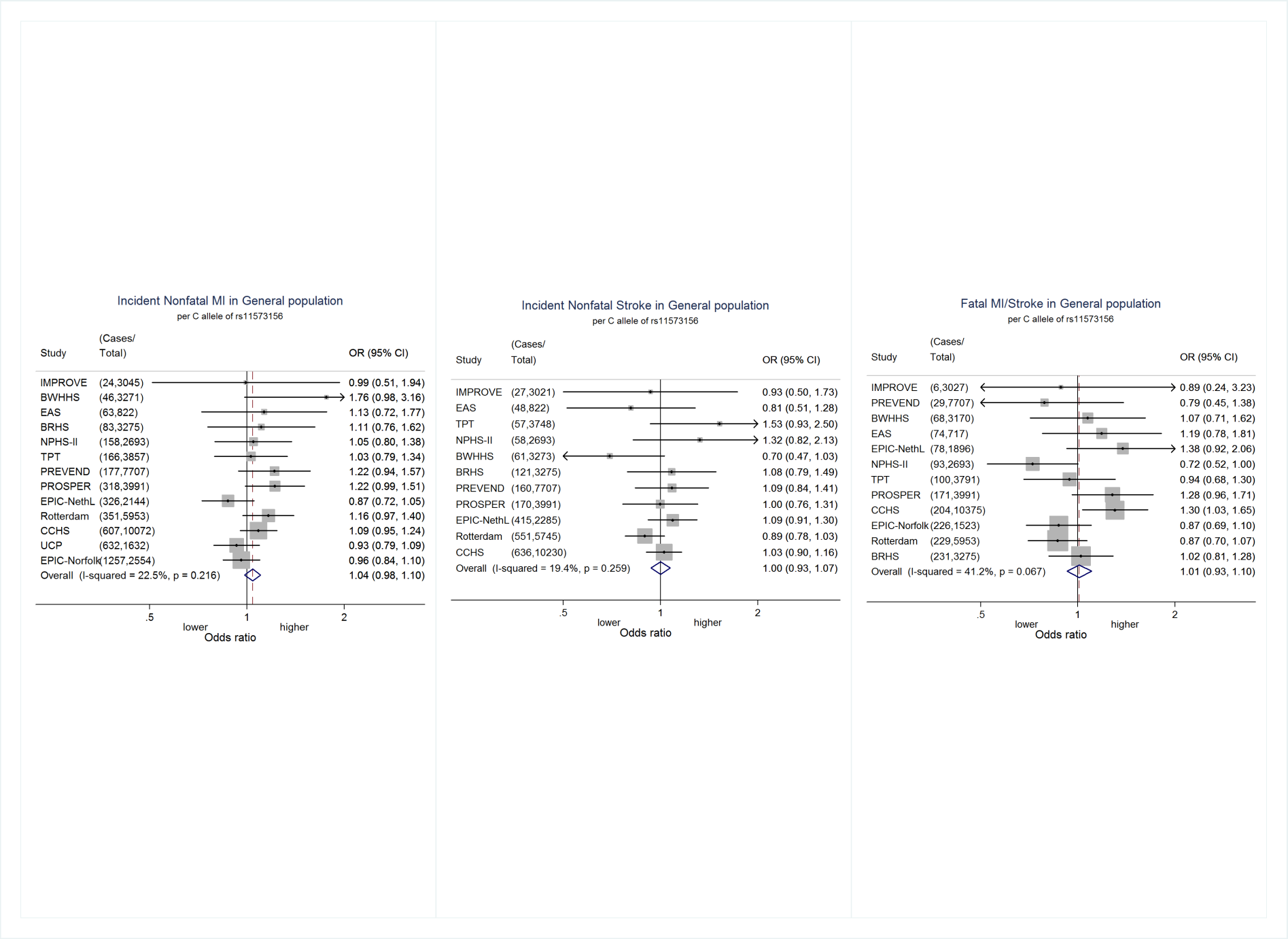
 **(b) (c)**

## Figure 9. Meta-analysis of the effect of *PLA2G2A* rs11573156 in general population studies and risk of: (a) prevalent myocardial infarction; (b) prevalent stroke, and; (c) prevalent myocardial infarction or angiographically-determined coronary artery disease.

**
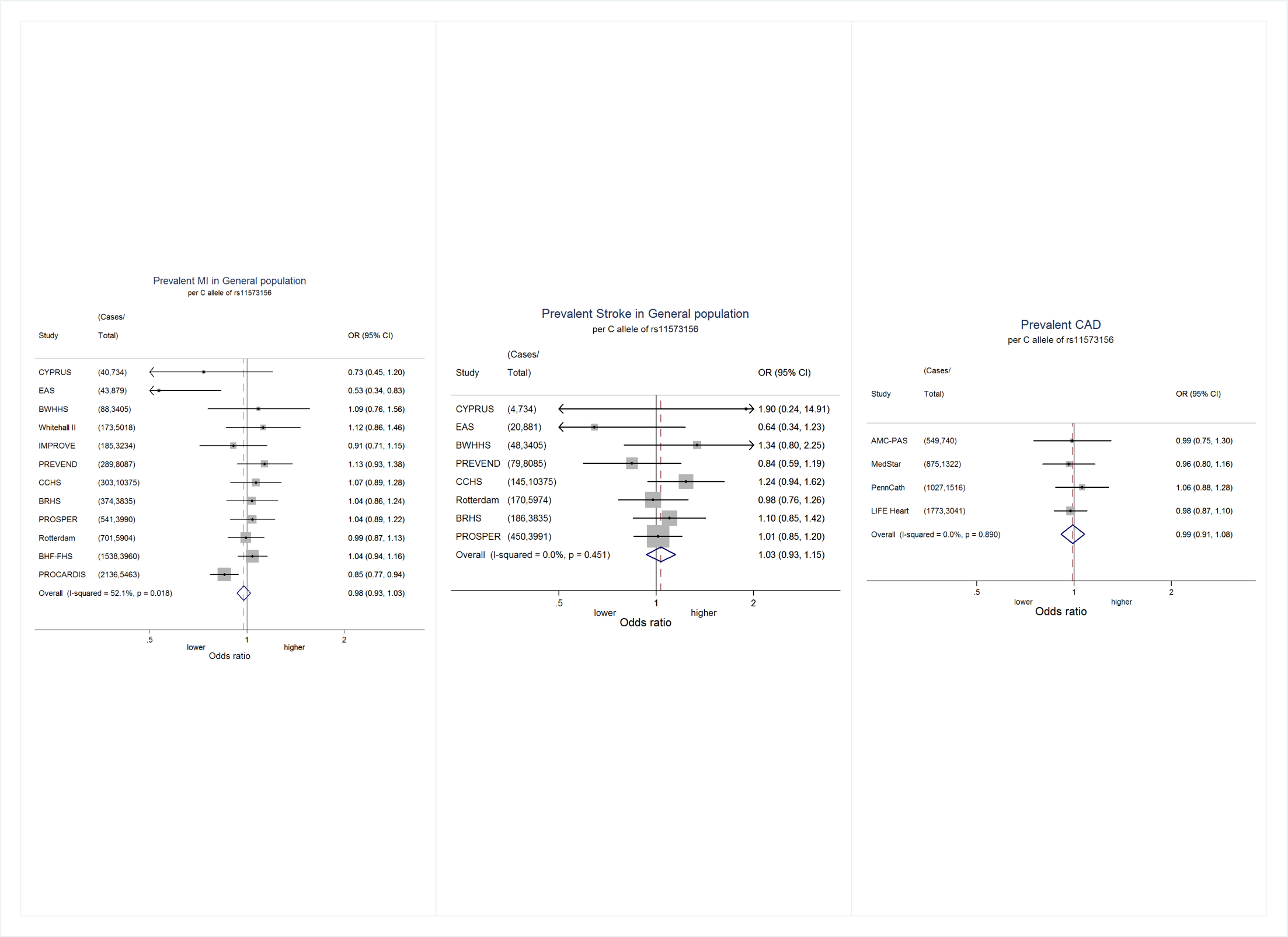
 (a) (b) (c)**

## Figure 10. Meta-analysis of the effect of *PLA2G2A* rs11573156 in studies of acute coronary syndrome and risk of: (a) incident nonfatal myocardial infarction (b) incident nonfatal stroke; (c) fatal myocardial infarction, stroke or all-cause mortality.

**(a) (b) (c)**

**
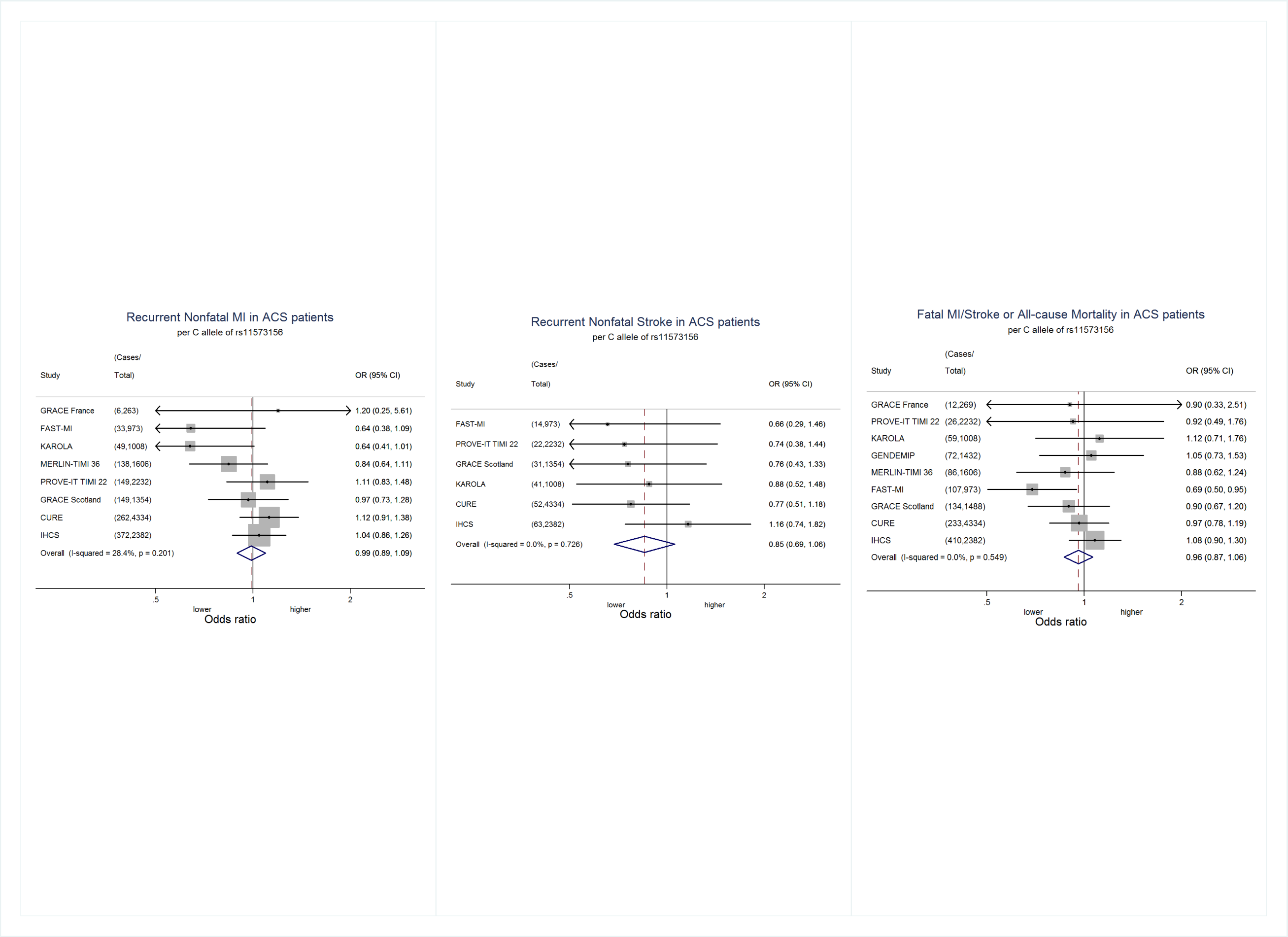
**

# Methods

## Association of circulating sPLA_2_ IIA-mass with sPLA_2_ enzyme activity

In studies that measured both sPLA_2_ traits (IIA mass and enzyme activity), we measured the cross-sectional association between log sPLA_2_-IIA mass and log sPLA_2_ enzyme activity using Pearson’s correlation coefficient.

## Association of circulating sPLA_2_ IIA-mass and sPLA_2_ enzyme activity with cardiovascular traits and clinical events

We evaluated the cross-sectional association between log sPLA_2_-IIA mass and log sPLA_2_ enzyme activity with the following traits: age, body mass index (BMI), C-reactive protein (CRP), systolic blood pressure (SBP), diastolic blood pressure (DBP), high density lipoprotein cholesterol (HDL-C), low density lipoprotein cholesterol (LDL-C), triglycerides (TG), apolipoprotein A1, apolipoprotein B, gender, type 2 diabetes (T2D) and smoking status. We firstly created tertiles of sPLA_2_-IIA mass and sPLA_2_ enzyme activity, and tabulated the mean and standard deviation (SD) for continuous traits and proportion of cases for binary traits for each tertile. Secondly, we performed univariate linear and logistic regression analyses for continuous and binary traits, respectively, using natural logarithm (log[e]) transformed sPLA_2_ IIA mass and sPLA_2_ enzyme activity as the explanatory (independent) variables.

To evaluate the shape of the association between sPLA_2_-IIA mass, sPLA_2_ enzyme activity and cardiovascular events, we used minimally-adjusted logistic regression models with log sPLA_2_-IIA mass and log sPLA_2_ enzyme activity as independent continuous variables (assuming a linear effect) and repeated the regression after adding a quadratic term to the linear one. We tested the null hypothesis, that the linear model explained the outcome better, by using the likelihood ratio test.

In order to investigate the robustness of the association between sPLA_2_ and fatal/nonfatal MI, we conducted a step-wise multivariate analysis. In the general population cohort (EPIC-Norfolk), we firstly created a minimally adjusted model between log sPLA_2_-IIA enzyme mass or log sPLA_2_ activity with MI, adjusted only for age and gender. We then added BMI, BP and T2D to the model (but we did not include lipids as they may mediate a potential association between sPLA_2_ and CVD). In the next model, we also adjusted for LDL-C, HDL-C and TG. Finally, in order to investigate the independent effect of sPLA_2_-IIA mass and sPLA_2_ enzyme activity with MI, we adjusted for the corresponding (non-index) trait. For the ACS cohorts, the covariates added to the minimally-adjusted model were BMI and BP (since not all traits were available in all studies).

## Genetic analyses

A pre-specified script was developed to standardize the genetic analysis in the collaborating studies. We limited analysis of associations between genotype and continuous traits to controls in retrospective case-control studies to avoid the potential for established vascular disease to alter the magnitude of the genotype-trait association. For cohort studies and nested case-control studies, we included all participants as measurement of variables was made prior to the clinical event.

## Association between genetic variant and cardiovascular events

In addition to the major vascular event outcome analysed and reported in the main manuscript, a further six studies, four in which the primary outcome was angiographically-determined coronary artery disease (AMC-PAS, LIFE, MedStar and PennCath) and two set in patients with established cardiovascular disease (SMART and GENDER), were analyzed and presented separately, as their patient characteristics or outcome definitions differed from other studies (see **eResults**).

## Instrumental variable analysis

We used the ratio instrumental variable (IV) estimator to estimate the unconfounded effect of log sPLA_2_-IIA mass and sPLA_2_ enzyme activity on cardiovascular outcomes. For this, we conducted a fixed-effects meta-analysis of rs11573156 on log sPLA_2_-IIA mass and log sPLA_2_ enzyme activity across studies with these phenotypes available, performed separately for general population and ACS studies. Assuming a single effect of rs11573156 on log sPLA_2_-IIA mass and log sPLA_2_ enzyme activity separately for general population and ACS studies, we applied the appropriate values to studies that did not have information on sPLA_2_. Using study-specific associations between rs115713156 and each outcome, we calculated the IV estimate for log sPLA_2_-IIA mass and log sPLA_2_ enzyme activity by dividing the rs11573156-outcome association by the pooled estimate of the association of rs11573156 and log sPLA_2_-IIA mass and sPLA_2_ enzyme activity. This analysis took into account the uncertainty in both rs11573156-sPLA_2_ and rs11573156-outcome associations by using the delta method to estimate the standard errors of ratio instrumental variable estimates. The study specific IV estimates were then pooled using fixed effects meta-analysis.

This IV technique allowed us to include studies that did not have measures of sPLA_2_, which should increase the precision of pooled IV estimates, but this may occur at a loss to the internal consistency of the IV estimate for studies without measures of sPLA_2_.

## Analysis of specificity of SNPs in *PLA2G2A* with mRNA expression of *PLA2G2A*, *PLA2G5* and *PLA2G10*

The Advanced Study of Aortic Pathology (ASAP) recruited patients undergoing aortic valve surgery at the Karolinska University Hospital, Stockholm Sweden. Tissue biopsies were taken from liver, heart, mammary arteries and dilated and non-dilated ascending aorta during surgery. A total of 700 tissue samples from 272 different patients were investigated. The medial and adventitial layers of the vascular specimen were isolated by adventicectomy. All tissue samples were incubated with RNA later (Ambion, Austin, Texas, USA) and homogenised for mRNA extraction as previously detailed. Affymetrix GeneChip Human Exon 1.0 ST expression arrays were used and raw data were pre-processed using the RMA algorithm and the core group of metaprobesets, resulting in normalized and log(2) transformed expression data. Gene expression and eQTL effects were evaluated as previously described. Briefly, genotypes were re-coded as 0, 1 and 2 and included as predictors in a linear additive model with gene expression as the response variable. All calculations were performed using R 2.13.0 and Bioconductor. Alignments and plots of exon-level microarray data were performed using the GeneRegionScan package.

We used an external data source comprising 206 transplant donor liver samples to replicate the SNP showing strongest association with *PLA2G2A* mRNA expression.

## Search Strategy: overview of randomized trials of the sPLA_2_-inhibitor varespladib on circulating sPLA_2_-IIA mass and cardiovascular risk factors

Our PubMed search text consisted of (“varespladib”[Substance Name] OR "varespladib methyl "[Substance Name] OR varespladib OR A002 OR LY333013). Studies that were randomized trials comparing varespladib to placebo were selected for inclusion. Data were extracted (by HJE) and a random subset was checked (by MVH); discrepancies were resolved by consensus. Where possible, we harmonised variable units between studies. In the case where a conversion factor could not be identified, we used the standardized mean difference.

## Outcome Definitions in Collaborating Studies

***General Population Studies***

*BHF-FHS*  **MI**: Direct assessment of hospital records or confirmation by a general practitioner (GP) of a documented MI

*BRHS* **MI**: Events were identified from GP records and confirmed with the GP using WHO criteria. **Stroke:** Events were identified from GP records and confirmed with GP (acute disturbance of cerebral function of presumed vascular origin lasting ≥24 hours).

*BWHHS* **MI/stroke**: Events were obtained from self-report, GP records and confirmed with the GP using WHO criteria. Fatal events were defined using the following ICD-10 codes: ICD10: I20-I25 and I60-I65.

*CCHS*  **MI**: ICD8: 410; ICD10: I21-I22; **Stroke**: ICD8: 432-434, ICD10: I63

*CYPRUS*  **MI**: based on clinical history and electrocardiogram (ECG) findings (which were centrally reported by a cardiologist and vascular internist); **Stroke**: Hospital records and self-report

*EAS* **MI**: AHA criteria or death certification recording ICD-10 codes: I20-I25. **Stroke**: Symptom onset ≤48 hours previously and lasting ≥24 hours, CT evidence of cerebral infarction or haemorrhage, or hospital discharge diagnosis or death certification recording ICD10: I60-I65.

*EPIC-Netherlands*  **MI**: ICD-9:410; ICD-10: I21, I22; **Stroke**: ICD-9:430-434, 436; ICD-10: I60-I66

*EPIC-Norfolk*  **MI**: ICD9 codes 410-414

*IMPROVE*  **MI:** diagnosed according to European Society for Cardiology guidelines; **Stroke**: diagnosed according to a validated score; using hospital records and death certificates.

*NPHSII* **MI**: ICD-9: 410; Stroke: ICD-9: 430-436

*PREVEND*  **MI**: Cardiac events were reviewed by a clinical event committee and divided into ST-segment elevation myocardial infarctions (STEMI) or non-ST-elevation acute coronary syndromes (ACS). ST-segment elevation MI was defined as chest pain and ST elevation >1 mm in at least two contiguous leads. Non-ST-elevation acute coronary syndrome was defined as chest pain with positive cardiac markers (troponin or creatinine kinase) and/or dynamic ST-segment changes (ICD-9 410, 411); **Fatal MI/stroke**: based on ICD-10 codes: I01-99

*PROCARDIS* **MI**: documentation of two or more of (a) typical ischaemic chest pain, pulmonary oedema, syncope or shock; (b) development of pathological Q-waves and/or appearance or disappearance of localized ST elevation followed by T-wave inversion in two or more standard electrocardiograph leads; (c) increase in concentration of serum enzymes consistent with MI (e.g. creatine kinase more than twice the upper limit of normal). **Symptomatic ACS**: documentation of hospitalization for one of the following indications: (a) unstable angina diagnosed by typical ischemic chest pain at rest associated with reversible ST-depression in two or more standard electrocardiograph leads; (b) thrombolysis for suspected MI (as indicated by localized ST-elevation in two or more standard electrocardiograph leads) even without later development of T-wave inversion, Q-waves, or a significant enzyme rise; or (c) emergency revascularization (i.e. during same admission) following presentation with typical ischemic chest pain at rest.

*PROSPER* **MI/stroke**: participants were monitored every 3 months for clinical events

*Rotterdam*  Information was obtained from general practitioners and discharge reports/letters from medical specialists. Two research physicians independently coded all reported **MI** and **stroke** events according to ICD-10 and a medical expert reviewed all events. Fatal events were defined as deaths related until 28 days after MI or stroke using ICD-10 codes: I20-I25, I46, R96 and I50.

*TPT*  For both **MI and Stroke,** diagnosis was by WHO criteria, and verified by an independent reviewer

*UCP* **MI**: ICD-9 code 410

*Whitehall II*  **MI/stroke** events were obtained from ICD-9 codes 390.0–458.9 and ICD-10 codes I00–I99.

***Acute Coronary Syndrome Cohorts***

*CURE* **MI**: recurrent MI was defined by the presence of at least two of the following: ischemic chest pain; elevation of serum levels of cardiac markers or enzymes (troponin, creatine kinase, creatine kinase MB isoenzyme, or other cardiac enzymes) to at least twice the upper limit of normal reference range or three times the upper limit of normal within 48 hours after percutaneous coronary intervention (or to a level 20% higher than the previous value if the level had already been elevated because of an early myocardial infarction); and electrocardiographic changes compatible with infarction. **Stroke** was defined as a new focal neurologic deficit of vascular origin lasting more than 24 hours. **Death** from cardiovascular causes was defined as any death for which there was no clearly documented nonvascular cause.

*FAST-MI* **MI:** Recurrent MI was defined as recurrent symptoms with a new rise in cardiac markers. Follow-up data were collected through contacts with the attending physicians, patients, or family. Vital status of missing participants was assessed from the registries of the patients’ birthplaces.

*GENDEMIP* **Fatal MI/stroke:**  mortality data were obtained from death certificates (ICD-10 codes I00-I99) by the Institute of Health Information and Statistics of the Czech Republic, which includes from the date of admission (2006-2009) to November 2011.

*GRACE Scotland/ France* **MI**: STEMI diagnosis was based on new ST-segment elevation >1 mm in any location, or if a new left-bundle-branch block was identified on ECG, with at least one positive cardiac biochemical marker of necrosis raised above the diagnostic threshold for infarction. Non-STEMI was diagnosed if the marker of necrosis was raised without ST-segment elevation on index or a subsequent ECG. Deaths were recorded during the index period (0–6 days) and subsequently (>6 days).

*IHCS* **MI/stroke**: information was obtained from physician report and hospital readmission for MI or stroke using ICD-9 codes.

*KAROLA* **MI/stroke**: Information was obtained from the primary care physician using a standardized questionnaire. For fatal events, information was obtained from the death certificate, and the main cause of death was coded according to the ICD-9. Secondary cardiovascular events were defined either as CVD as the main cause of death (as stated in the death certificate), nonfatal myocardial infarction (MI), or ischemic stroke.

*MERLIN-TIMI36* **MI**: criteria adapted from American College of Cardiology (including symptoms suggestive of ischemia/infarction associated with ECG, cardiac biomarker or pathological evidence of infarction. Outcomes were adjudicated by a clinical events committee.

*MIRACL* **MI**: cardiac enzyme and ECG data using NOVOCODE classification.

*PROVE-IT TIMI 22*  **MI**: Myocardial infarction was defined by the presence of symptoms suggestive of ischemia or infarction, with either electrocardiographic evidence (new Q waves in two or more leads) or cardiac-marker evidence of infarction, according to the standard TIMI and American College of Cardiology definition.

***Other Studies***

*AMC-PAS*  **MI/CAD**: MI, surgical or percutaneous revascularisation, coronary angiograph with ≥70% stenosis in a major epicardial artery

*GENDER* **Restenosis**: defined as clinical restenosis (comprising death, MI and target vessel revascularisation) within 9 months. All outcomes were evaluated by an independent committee.

*LIFE Heart*  **MI/CAD**: AMI or ≥50% stenosis on coronary angiography

*MedStar* **CAD**: ≥50% stenosis on coronary angiography

*PennCath* **MI/CAD**: AMI or ≥50% stenosis on coronary angiography

*SMART* **MI/Stroke** information from hospitalizations and outpatient clinic visits was obtained from participants by 6-monthly questionnaire. If a possible event was reported by participants, all available relevant data were collected. Death was reported by relatives, the GP or the specialist who treated the participant. All events were classified independently by committee, comprising physicians from different departments.

# Results

## Correlation between sPLA_2_-IIA mass and sPLA_2_ enzyme activity

We investigated the correlation between sPLA_2_-IIA mass and sPLA_2_ enzyme activity in three ACS cohorts and one general population cohort that reported information on both sPLA_2_-IIA mass and sPLA_2_ enzyme activity. The Pearson’s correlation coefficients between sPLA_2_-IIA mass and sPLA_2_ enzyme activity in the ACS studies were 0.50, 0.51 and 0.58 whereas for the general population study, it was 0.18 (all P values for correlation <0.0001) (**eFigure 2**).

## Observational analysis of sPLA_2_-IIA mass and sPLA_2_ enzyme activity with cardiovascular events

sPLA_2_ enzyme activity showed independent association with fatal/nonfatal MI in the general population study and nonfatal MI/all-cause mortality in ACS studies when adjusted for sPLA_2_-IIA mass and potential confounders (**Figure 1**). A regression analysis conducted to determine the shape of the association between sPLA_2_-IIA mass and sPLA_2_ enzyme activity with cardiovascular outcomes showed a linear model as the best fit in each study analysed (**eTable 6**).

## Association between *PLA2G2A* rs11573156 and other cardiovascular events

In a meta-analysis including 4 studies (4224 cases in 6619 participants), no association between the C allele of rs11573156 was identified for angiographically-determined coronary artery disease (OR 0.99; 95%CI: 0.91, 1.08; **eFigure 9c**). In a study set in patients with established vascular disease or high cardiovascular risk (SMART), the association between the C allele of rs11573156 with recurrent MVE (fatal/non-fatal MI or stroke) was OR 0.92 (95%CI: 0.80, 1.07). For the study set in patients with ACS undergoing PCI (GENDER), the association between the C allele of rs11573156 with coronary artery restenosis was OR 1.04 (95%CI: 0.82, 1.31).

# References

1. Samani NJ, Burton P, Mangino M et al. A genomewide linkage study of 1,933 families affected by premature coronary artery disease: The British Heart Foundation (BHF) Family Heart Study. Am J Hum Genet 2005;77:1011-20.

2. Shaper AG, Pocock SJ, Walker M, Cohen NM, Wale CJ, Thomson AG. British Regional Heart Study: cardiovascular risk factors in middle-aged men in 24 towns. Br Med J (Clin Res Ed) 1981;283:179-86.

3. Lawlor DA, Bedford C, Taylor M, Ebrahim S. Geographical variation in cardiovascular disease, risk factors, and their control in older women: British Women's Heart and Health Study. J Epidemiol Community Health 2003;57:134-40.

4. Nordestgaard BG, Agerholm-Larsen B, Wittrup HH, Tybjaerg-Hansen A. A prospective cardiovascular population study used in genetic epidemiology. The Copenhagen City Heart Study. Scand J Clin Lab Invest Suppl 1996;226:65-71.

5. Panayiotou A, Nicolaides A, Griffin M et al. Serum total homocysteine, folate, 5,10-methylenetetrahydrofolate reductase (MTHFR) 677C-->T genotype and subclinical atherosclerosis. Expert Opin Ther Targets 2009;13:1-11.

6. Fowkes FG, Housley E, Cawood EH, Macintyre CC, Ruckley CV, Prescott RJ. Edinburgh Artery Study: prevalence of asymptomatic and symptomatic peripheral arterial disease in the general population. Int J Epidemiol 1991;20:384-92.

7. Beulens JW, Monninkhof EM, Verschuren WM et al. Cohort profile: the EPIC-NL study. Int J Epidemiol 2010;39:1170-8.

8. Day N, Oakes S, Luben R et al. EPIC-Norfolk: study design and characteristics of the cohort. European Prospective Investigation of Cancer. Br J Cancer 1999;80 Suppl 1:95-103.

9. Tobin MD, Raleigh SM, Newhouse S et al. Association of WNK1 gene polymorphisms and haplotypes with ambulatory blood pressure in the general population. Circulation 2005;112:3423-9.

10. Baldassarre D, Nyyssonen K, Rauramaa R et al. Cross-sectional analysis of baseline data to identify the major determinants of carotid intima-media thickness in a European population: the IMPROVE study. Eur Heart J 2010;31:614-22.

11. Miller GJ, Bauer KA, Barzegar S, Cooper JA, Rosenberg RD. Increased activation of the haemostatic system in men at high risk of fatal coronary heart disease. Thromb Haemost 1996;75:767-71.

12. Diercks GF, Janssen WM, van Boven AJ et al. Rationale, design, and baseline characteristics of a trial of prevention of cardiovascular and renal disease with fosinopril and pravastatin in nonhypertensive, nonhypercholesterolemic subjects with microalbuminuria (the Prevention of REnal and Vascular ENdstage Disease Intervention Trial [PREVEND IT]). Am J Cardiol 2000;86:635-8.

13. PROCARDIS Consortium. A trio family study showing association of the lymphotoxin-alpha N26 (804A) allele with coronary artery disease. Eur J Hum Genet 2004;12:770-4.

14. Shepherd J, Blauw GJ, Murphy MB et al. Pravastatin in elderly individuals at risk of vascular disease (PROSPER): a randomised controlled trial. Lancet 2002;360:1623-30.

15. Hofman A, Breteler MM, van Duijn CM et al. The Rotterdam Study: objectives and design update. Eur J Epidemiol 2007;22:819-29.

16. Meade TW, Wilkes HC, Stirling Y, Brennan PJ, Kelleher C, Browne W. Randomized controlled trial of low dose warfarin in the primary prevention of ischaemic heart disease in men at high risk: design and pilot study. Eur Heart J 1988;9:836-43.

17. van Wieren-de Wijer DB, Maitland-van der Zee AH, de Boer A et al. Recruitment of participants through community pharmacies for a pharmacogenetic study of antihypertensive drug treatment. Pharm World Sci 2009;31:158-64.

18. Dhamrait SS, Stephens JW, Cooper JA et al. Cardiovascular risk in healthy men and markers of oxidative stress in diabetic men are associated with common variation in the gene for uncoupling protein 2. Eur Heart J 2004;25:468-75.

19. Marmot M, Brunner E. Cohort Profile: the Whitehall II study. Int J Epidemiol 2005;34:251-6.

20. Yusuf S, Zhao F, Mehta SR, Chrolavicius S, Tognoni G, Fox KK. Effects of clopidogrel in addition to aspirin in patients with acute coronary syndromes without ST-segment elevation. N Engl J Med 2001;345:494-502.

21. Cambou JP, Simon T, Mulak G, Bataille V, Danchin N. The French registry of Acute ST elevation or non-ST-elevation Myocardial Infarction (FAST-MI): study design and baseline characteristics. Arch Mal Coeur Vaiss 2007;100:524-34.

22. Pitha J, Hubáček JA, Poledne R et al. Genetic determination of the prognosis in survivors of acute coronary syndromes. Study design and rationale for a multicenter study. Cor et Vasa 2007;49:134-137.

23. Rationale and design of the GRACE (Global Registry of Acute Coronary Events) Project: a multinational registry of patients hospitalized with acute coronary syndromes. Am Heart J 2001;141:190-9.

24. Granger CB, Goldberg RJ, Dabbous O et al. Predictors of hospital mortality in the global registry of acute coronary events. Arch Intern Med 2003;163:2345-53.

25. Zebrack JS, Anderson JL, Maycock CA, Horne BD, Bair TL, Muhlestein JB. Usefulness of high-sensitivity C-reactive protein in predicting long-term risk of death or acute myocardial infarction in patients with unstable or stable angina pectoris or acute myocardial infarction. Am J Cardiol 2002;89:145-9.

26. Rothenbacher D, Koenig W, Brenner H. Comparison of N-terminal pro-B-natriuretic peptide, C-reactive protein, and creatinine clearance for prognosis in patients with known coronary heart disease. Arch Intern Med 2006;166:2455-60.

27. Morrow DA, Scirica BM, Karwatowska-Prokopczuk E et al. Effects of ranolazine on recurrent cardiovascular events in patients with non-ST-elevation acute coronary syndromes: the MERLIN-TIMI 36 randomized trial. JAMA 2007;297:1775-83.

28. Ryu SK, Mallat Z, Benessiano J et al. Phospholipase A2 enzymes, high-dose atorvastatin, and prediction of ischemic events after acute coronary syndromes. Circulation 2012;125:757-66.

29. Cannon CP, Braunwald E, McCabe CH et al. Intensive versus moderate lipid lowering with statins after acute coronary syndromes. N Engl J Med 2004;350:1495-504.

30. Trip MD, Smulders YM, Wegman JJ et al. Frequent mutation in the ABCC6 gene (R1141X) is associated with a strong increase in the prevalence of coronary artery disease. Circulation 2002;106:773-5.

31. Folkersen L, van't Hooft F, Chernogubova E et al. Association of genetic risk variants with expression of proximal genes identifies novel susceptibility genes for cardiovascular disease. Circ Cardiovasc Genet 2010;3:365-73.

32. Sampietro ML, Pons D, de Knijff P, Slagboom PE, Zwinderman A, Jukema JW. A genome wide association analysis in the GENDER study. Neth Heart J 2009;17:262-4.

33. Beutner F, Teupser D, Gielen S et al. Rationale and design of the Leipzig (LIFE) Heart Study: phenotyping and cardiovascular characteristics of patients with coronary artery disease. PLoS One 2011;6:e29070.

34. Reilly MP, Li M, He J et al. Identification of ADAMTS7 as a novel locus for coronary atherosclerosis and association of ABO with myocardial infarction in the presence of coronary atherosclerosis: two genome-wide association studies. Lancet 2011;377:383-92.

35. Simons PC, Algra A, van de Laak MF, Grobbee DE, van der Graaf Y. Second manifestations of ARTerial disease (SMART) study: rationale and design. Eur J Epidemiol 1999;15:773-81.

36. Wolbink GJ, Schalkwijk C, Baars JW, Wagstaff J, van den Bosch H, Hack CE. Therapy with interleukin-2 induces the systemic release of phospholipase-A2. Cancer Immunol Immunother 1995;41:287-92.

37. Nevalainen TJ, Eerola LI, Rintala E, Laine VJ, Lambeau G, Gelb MH. Time-resolved fluoroimmunoassays of the complete set of secreted phospholipases A2 in human serum. Biochim Biophys Acta 2005;1733:210-23.

38. Radvanyi F, Jordan L, Russo-Marie F, Bon C. A sensitive and continuous fluorometric assay for phospholipase A2 using pyrene-labeled phospholipids in the presence of serum albumin. Anal Biochem 1989;177:103-9.

39. Pernas P, Masliah J, Olivier JL, Salvat C, Rybkine T, Bereziat G. Type II phospholipase A2 recombinant overexpression enhances stimulated arachidonic acid release. Biochem Biophys Res Commun 1991;178:1298-305.

40. Holdt LM, Thiery J, Breslow JL, Teupser D. Increased ADAM17 mRNA expression and activity is associated with atherosclerosis resistance in LDL-receptor deficient mice. Arterioscler Thromb Vasc Biol 2008;28:1097-103.

41. Rosenson RS, Hislop C, Elliott M, Stasiv Y, Goulder M, Waters D. Effects of varespladib methyl on biomarkers and major cardiovascular events in acute coronary syndrome patients. J Am Coll Cardiol 2010;56:1079-88.

42. Rosenson RS, Hislop C, McConnell D et al. Effects of 1-H-indole-3-glyoxamide (A-002) on concentration of secretory phospholipase A2 (PLASMA study): a phase II double-blind, randomised, placebo-controlled trial. Lancet 2009;373:649-58.

43. Rosenson RS, Elliott M, Stasiv Y, Hislop C. Randomized trial of an inhibitor of secretory phospholipase A2 on atherogenic lipoprotein subclasses in statin-treated patients with coronary heart disease. Eur Heart J 2011;32:999-1005.

44. Dzavik V, Lavi S, Thorpe K et al. The sPLA2 Inhibition to Decrease Enzyme Release after Percutaneous Coronary Intervention (SPIDER-PCI) trial. Circulation 2010;122:2411-8.

45. Snyder DW, Bach NJ, Dillard RD et al. Pharmacology of LY315920/S-5920, [[3-(aminooxoacetyl)-2-ethyl-1- (phenylmethyl)-1H-indol-4-yl]oxy] acetate, a potent and selective secretory phospholipase A2 inhibitor: A new class of anti-inflammatory drugs, SPI. J Pharmacol Exp Ther 1999;288:1117-24.

46. Fraser H, Hislop C, Christie RM et al. Varespladib (A-002), a secretory phospholipase A2 inhibitor, reduces atherosclerosis and aneurysm formation in ApoE-/- mice. J Cardiovasc Pharmacol 2009;53:60-5.

47. Holdt LM, Beutner F, Scholz M et al. ANRIL expression is associated with atherosclerosis risk at chromosome 9p21. Arterioscler Thromb Vasc Biol 2010;30:620-7.

48. Thomas DC, Lawlor DA, Thompson JR. Re: Estimation of bias in nongenetic observational studies using "Mendelian triangulation" by Bautista et al. Ann Epidemiol 2007;17:511-3.

49. Angrist JD, Krueger AB. Split-Sample Instrumental Variables Estimates of the Return to Schooling. Journal of Business & Economic Statistics 1995;13:225-35.

50. Thompson JR, Minelli C, Abrams KR, Tobin MD, Riley RD. Meta-analysis of genetic studies using Mendelian randomization--a multivariate approach. Stat Med 2005;24:2241-54.

51. Inoue A, Solon G. Two-Sample Instrumental Variables Estimators. Review of Economics and Statistics 2010;92:557-561.

52. Irizarry RA, Hobbs B, Collin F et al. Exploration, normalization, and summaries of high density oligonucleotide array probe level data. Biostatistics 2003;4:249-64.

53. Gentleman RC, Carey VJ, Bates DM et al. Bioconductor: open software development for computational biology and bioinformatics. Genome Biol 2004;5:R80.

54. Folkersen L, Diez D, Wheelock CE et al. GeneRegionScan: a Bioconductor package for probe-level analysis of specific, small regions of the genome. Bioinformatics 2009;25:1978-9.

55. Innocenti F, Cooper GM, Stanaway IB et al. Identification, replication, and functional fine-mapping of expression quantitative trait loci in primary human liver tissue. PLoS Genet 2011;7:e1002078.

56. Brott T, Adams HP, Jr., Olinger CP et al. Measurements of acute cerebral infarction: a clinical examination scale. Stroke 1989;20:864-70.

57. Rautaharju PM, Calhoun HP, Chaitman BR. NOVACODE serial ECG classification system for clinical trials and epidemiologic studies. J Electrocardiol 1992;24 Suppl:179-87.
